# Differential Labeling of Chemically Modified Peptides and Lipids among Cyanobacteria *Planktothrix* and *Microcystis*

**DOI:** 10.3390/microorganisms9081578

**Published:** 2021-07-24

**Authors:** Rubén Morón-Asensio, David Schuler, Anneliese Wiedlroither, Martin Offterdinger, Rainer Kurmayer

**Affiliations:** 1 Research Department for Limnology, University of Innsbruck, Mondseestrasse 9, 5310 Mondsee, Austria; david.schuler@uibk.ac.at (D.S.); anneliese.wiedlroither@uibk.ac.at (A.W.); 2 Core Facility Biooptics (CCB), Medical University Innsbruck, Innrain 80-82, 6020 Innsbruck, Austria; martin.offterdinger@i-med.ac.at

**Keywords:** cyanotoxin, microcystin, anabaenopeptin, CuAAC, biorthogonal, lipids, BODIPY, ALEXA, colocalization

## Abstract

The cyanoHAB forming cyanobacteria *Microcystis* and *Planktothrix* frequently produce high intracellular amounts of microcystins (MCs) or anabaenopeptins (APs). In this study, chemically modified MCs and APs have been localized on a subcellular level in *Microcystis* and *Planktothrix* applying copper-catalyzed alkyne-azide cycloaddition (CuACC). For this purpose, three different non-natural amino acids carrying alkyne or azide moieties were fed to individual *P. agardhii* strains No371/1 and CYA126/8 as well as to *M. aeruginosa* strain Hofbauer showing promiscuous incorporation of various amino acid substrates during non-ribosomal peptide synthesis (NRPS). Moreover, CYA126/8 peptide knock-out mutants and non-toxic strain *Synechocystis* PCC6803 were processed under identical conditions. Simultaneous labeling of modified peptides with ALEXA405 and ALEXA488 and lipid staining with BODIPY 505/515 were performed to investigate the intracellular location of the modified peptides. Pearson correlation coefficients (PCC) obtained from confocal images were calculated between the different fluorophores and the natural autofluorescence (AF), and between labeled modified peptides and dyed lipids to investigate the spatial overlap between peptides and the photosynthetic complex, and between peptides and lipids. Overall, labeling of modified MCs (*M. aeruginosa*) and APs (*P. agardhii*) using both fluorophores revealed increased intensity in MC/AP producing strains. For *Synechocystis* lacking NRPS, no labeling using either ALEXA405 or ALEXA488 was observed. Lipid staining in *M. aeruginosa* and *Synechocystis* was intense while in *Planktothrix* it was more variable. When compared with AF, both modified peptides and lipids showed a heterologous distribution. In comparison, the correlation between stained lipids and labeled peptides was not increased suggesting a reduced spatial overlap.

## 1. Introduction

Planktonic toxin-producing cyanobacteria of the genera *Microcystis* and *Planktothrix* frequently form algal blooms in freshwater systems. The accumulation of cyanobacterial biomass due to cyanobacterial harmful algal blooms (CHABs) and their toxic or bioactive metabolites can cause diseases or even death of animals drinking the polluted waters. This can also be harmful to people if the blooms are produced in drinking water sources, and it may be due to their prolific secondary metabolism why cyanobacteria increase particularly in habitats influenced by eutrophication.

The microcystins (MCs) and anabaenopeptins (APs) are among the most common peptides produced by cyanobacteria. These two peptide families are synthesized via non-ribosomal peptide synthesis (NRPS) by large multifunctional enzyme complexes, using partly non-proteinogenic amino acids as substrates [1]. MCs are well known for their toxicity as inhibitors of protein-phosphatase 1 and 2A in the nanomolar range. APs have been shown to act as bioactive peptides as well, due to their capability of inhibiting proteases such as carboxypeptidase A [2].

Although MCs and APs are generally well described, yet their intracellular storage and release to the environment are not well understood. In general, according to the genetic basis, both MCs and APs are exported through the inner periplasmatic membrane via a dedicated ABC transporter forming an integrative part of the operon [3,4]. Alternatively, a passive release mechanism due to cell lysis and apoptosis has been investigated [5]. NRPS synthesis is encoded by several genes organized in clusters, i.e., *mcy*ABCDEGHIJ for MCs and *apn*ABCD for APs, respectively. Both clusters contain genes annotated as ABC transporters (*mcyH* and *apnD*) suggesting active transport and extracellular release of the metabolite.

Notably, the intracellular MC or AP content can be high, possibly approaching a solubility threshold inside the cell. Therefore, it has been hypothesized that an active storage mechanism or organelles supporting storage are likely to exist [6,7]. In particular, proteomic analysis has shown a chemical binding of MCs carrying the Mdha moiety to free cysteine residues of abundant intracellular proteins such as phycobilin and Rubisco [7,8]. It has been also observed earlier that the hydrophobic Adda moiety from MCs may interact with membranes and in fact nodularin, an analogue to MCs, has been shown to be able to form pores in lipid bilayers and natural membranes [9]. By means of electronic tomography spatial distribution of lipids inside the cells of the model cyanobacterium *Synechocystis* PCC6803 has been monitored and was found to be rather heterogeneous [10]. It is hypothesized that MCs and APs interact with lipids supporting their solubility.

Previous studies using immunogold labeling and cryosectioning have detected MCs associated with the thylakoid membrane, but the required sample preparation steps for electron microscopy may have misallocated the peptides [11]. Therefore, less invasive sample preparation tools are required for the intracellular detection and quantification of MCs and APs. For this purpose, non-natural alkyne and azide containing amino acids are applied as a substrate to different cyanobacterial strains with promiscuous adenylation domains in order to specifically tag MCs and APs molecules using endogenous biosynthesis via the respective NRPS [1,12].

**Figure 1:**
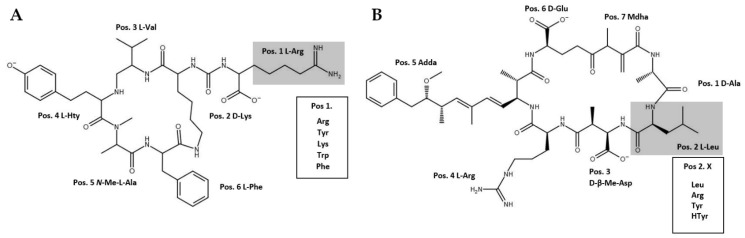
Naturally occurring (**A**) anabaenopeptin and (**B**) microcystin structural variants. Variable positions 1 in AP molecules and 2 in MC molecules are highlighted. The corresponding amino acids which may occur in these positions as observed in study organisms are shown in boxes.

Azide and alkyne groups do not occur endogenously and therefore are less likely to interact with other molecules inside the cytoplasm [13,14]. Natural mutations affecting certain adenylation domains of the respective NRPS pathway lead to a more promiscuous activation of the substrate allowing the incorporation of non-natural amino acids into the synthesized MCs and APs (Figure 1) [4,15,16] enabling targeting those molecules, i.e., with azide and alkyne carrying fluorophores through a “copper catalyzed azide-alkyne cycloaddition” (CuACC) [12,13].

In this study, we aim to further develop this technique by comparing AP/MC chemical modification and subsequent labeling using *Planktothrix agardhii* strain CYA126/8 and its engineered peptide knock out mutants [4,17,18]. By using this experimental setting, on the one hand, we aim to support the specificity and reproducibility of subcellular peptide labeling technique and on the other hand, we aim to increase our understanding of NRPS secondary metabolic pathways in general. Based on our previous study [12] we show novel chemical modification of APs/MCs through the integration of the non-natural AA L-4-azidophenylalanine (Phe-Az) or O-propargyl-L-Tyrosine (Prop-Tyr) as well as its consequences for fluorescence labeling. In addition to the green fluorophore applied previously a second fluorophore applied in the blue light spectrum has been tested to enable differential labeling of peptides and other organelles in the cell. As a proof of concept lipids were labeled using established dyes via green fluorescence and modified peptides were co-localized on a subcellular level.

## 2. Materials and Methods

### 2.1. Study Organisms

*P. agardhii* strain No371/1 (isolated from Moose Lake, Alberta, Canada by Rainer Kurmayer in 2005) and *P. agardhii* (NIVA-)CYA126/8 (isolated from Lake Langsjön, Stockholm, Sweden by Olav Skulberg in 1984) are known to carry a promiscuous adenylation domain ApnAA1, leading to the production of structural AP variants with variable amino acids occurring in the exocyclic position 1 [2]. *P. agardhii* CYA126/8 mutants with experimentally inactivated AP synthesis (ΔapnC), cyanopeptolin synthesis (ΔociA), microviridin synthesis (ΔmvdC), and microcystin synthesis (ΔmcyD) were included. Those knock out mutants have been generated earlier during the elucidation of the respective peptide synthesis pathways [4,17,18,19,20]. In order to reduce genetic variability, strains were regrown from one filament according to the standard filament isolation technique on agar [21]. Moreover, the MC-producing *Microcystis aeruginosa* strain Hofbauer (isolated from Lake Neusiedl, Burgenland, Niederösterreich, Austria by Barbara Hofbauer in 1982) was included for labeling MC structural variants. Finally, the strain *Synechocystis* PCC6803, which lacks NRPS coding genes [22], was included to test the specificity of the non-natural amino acid incorporation in general.

### 2.2. Growth Conditions

The strains were cultured in BG11 medium [21] at 20 °C and 40–60 µE m^−2^ s^−1^ semi-continuously following the turbidostat principle [23]. For the four gene inactivation mutants 1 µg mL^−1^ of chloramphenicol was added as a selection marker. During experimental feeding of non-natural amino acids, no further chloramphenicol was added. Precultures were started from cultures maintained at maximum growth rate conditions until they reached an optical density (OD_600nm_) of 0.1 (1 cm light path), then diluted to an OD_600nm_ of 0.01 and supplemented once either with 50 µM L-4-azidophenylalanine (Phe-Az), (Carl Roth, Karlsruhe, Germany), *N*-propargyloxy-carbonyl-L-lysine (Prop-Lys), (Sichem, Bremen, Germany) or O-propargyl-L-Tyrosine (Prop-Tyr) (Iris Biotech, Marktredwitz, Germany) dissolved in 1 mM NaOH. Control cultures were supplied with 1 mM NaOH only. Cells were finally collected after reaching an OD_600nm_ 0.1 (which was after 6 days for *M. aeruginosa* and *P. agardhii* and after 3 days for *Synechocystis* PCC6803). Three culture flasks per strain were inoculated under identical conditions, which were intended for the measuring of OD_600nm_, the labeling of the bioactive peptides and the peptide analysis through HPLC-MS (Figure S1).

### 2.3. Cell Fixation and Labeling

In brief, cells were harvested by centrifugation and fixed with 2% paraformaldehyde (PFA), permeated using Triton X-100 (0.1%, *v*/*v*) and washed with phosphate buffered saline (PBS). Click-chemical labeling was performed using two different fluorophores: blue fluorophore ALEXA405 azide (Click Chemistry Tools, Scottsdale, AZ, USA), and green fluorophore ALEXA488 azide/alkyne (Invitrogen, Thermo Fisher Scientific, Darmstadt, Germany). Since ALEXA405 was only available as an azide but not as an alkyne it could not be applied for MC or AP molecule labeling carrying the Phe-Az moiety. The general neutral lipid-binding fluorophore BODIPY 505/515 (4,4-difluoro-1,3,5,7-tetramethyl-4-bora-3a,4a-diaza-s-indacene) was obtained from Invitrogen (Thermo Fisher Scientific, Darmstadt, Germany). ALEXA fluorophores carrying alkyne or azide groups were used for targeting modified MCs or APs carrying non-natural amino acids whereas BODIPY 505/515 was applied for staining neutral lipids [24].

In general, cells were sedimented by centrifugation (14,000× *g*), washed with fresh PBS (3×), and fixed with 2% PFA (15 min). Samples were then washed again with PBS (3×) and permeated by incubating them for 10 min in PBS containing Triton X-100 (0.1%, *v*/*v*) and washed with PBS (3×). The resulting pellets were resuspended in 50 µL fresh PBS and stored at 4 °C.

For click-chemistry labeling, all cells were processed under identical conditions, the lipids were labeled through modified Brennan’s protocol [25,26]. In brief, cells were incubated in PBS at 37 °C for 10 min followed by a 10 min incubation with 4 µM BODIPY 505/515 at RT in the dark and washing excess of the fluorophore with PBS. Consecutively, the modified bioactive peptides were labeled using copper-catalyzed azide-alkyne cycloaddition (CuAAC) click reaction chemistry [13]. The cells were washed with 2% bovine serum albumin (BSA) while the reaction was performed according to manufactures instructions (Thermo Fisher Scientific Click-It^®^ Cell Reaction Buffer Kit), i.e., in a Tris-buffered reaction mix with 1 mM CuSO_4_ and various additives [13]. Supplemented blue fluorophore ALEXA405-azide or green fluorophore ALEXA488-azide were used to label modified peptides carrying alkyne moieties Prop-Tyr and Prop-Lys. Vice versa modified peptides carrying Phe-Az were detected via ALEXA488-alkyne. The concentrations at which the fluorophores were supplemented into the labeling reaction mix were 40 µM and 4 µM for ALEXA405-azide and ALEXA488-azide/alkyne, respectively. The reaction mix was incubated for 1 h in the dark and washed with BSA 2% and resuspended in 50 µL fresh PBS [12].

All strains were grown in the absence and presence of the three non-natural amino acids (Phe-Az, Prop-Lys and Prop-Tyr). Controls were made from cells grown in the absence of amino acids but treated under identical conditions. In addition, all treatments were processed and inspected under the confocal microscope without fluorophore addition. Finally, 5 µL of the samples were mounted using antifade solution (ProLong^®^ Diamond Antifade Mountant, Thermo Fisher Scientific, Darmstadt, Germany). The samples were air-dried for 48 h in the dark and stored at 4 °C until microscopical analysis.

### 2.4. Peptide Extraction

In parallel to cell preparation for labeling, cells were harvested via filtration using pre-weighed glass fiber (GF/C) filters. The filters with the collected biomass were dried using a vacuum centrifuge at RT for 4 h. The peptide content from the strains was extracted from the dried biomass using aqueous methanol (50% *v*/*v*) as described previously [27].

### 2.5. HPLC-MS Analysis

MCs and APs were separated by HPLC (HP 1100, Agilent, Vienna, Austria) system, using a linear mobile phase of water/acetonitrile (0.05% trifluoroacetic acid) gradient from 80:20 to 50:50 in 45 min at a flow rate of 1 mL/min through a LiChroCART 250-4 cartridge system (Merck, Darmstadt, Germany) with LiChrospher 100 octyldecyl silane (ODS), (5 µm particle size) as the solid reversed phase [12]. The HPLC system was coupled to an ESI-MS (Electrospray Ionization Mass Spectrometer) ion trap (amaZon SL Ion Trap MS, Bruker Daltonik, Bremen, Germany) operated in positive ionization mode. A mixture of nitrogen and helium was used as sheath gas and collision gas, respectively (43 psi, 9 L/min, 250 °C) with 5 kV capillary voltage. MC and AP variants were assigned according to their retention time, protonated mass [M+H]^+^, and fragmentation patterns. Fragmentation was achieved by automated fragmentation and adjusted to MS² for the two most abundant molecules showing the highest intensity while for MS³ only the peak with maximum intensity was further fragmented. LC-MS chromatograms and fragmentation patterns were investigated using the Bruker Compass data analysis software (version 4.2), (Bruker Daltonik, Bremen, Germany). Under these conditions the limit of detection for analytical standards MC-RR [M+H]^+^ 1038.5, MC-YR [M+H]^+^ 1045.5, and MC-LR [M+H]^+^ 995.5 was 10 ng injected (Cyanobiotech, Berlin, Germany).

### 2.6. Microscopic Analysis

Confocal images were acquired on a SP8 laser scanning microscope (Leica Microsystems, Wetzlar, Germany) at the Biooptics facilites (CCB) from *Medizinische Universität* Innsbruck. Images were acquired at an XY resolution of 50 nm and Z resolution of 150 nm. A total of 20 individual cells (*Microcystis* or *Synechocystis*) or filaments (*Planktothrix*) were randomly selected per strain and treatment. Confocal images were deconvolved to improve final resolution with Huygens Essential 20.04 software using the Classic Maximum Likelihood Estimation (CMLE) algorithm according to the manufacturer’s recommendations (Science Volume Imaging BV, Hilversum, The Netherlands).

**Figure 2:**
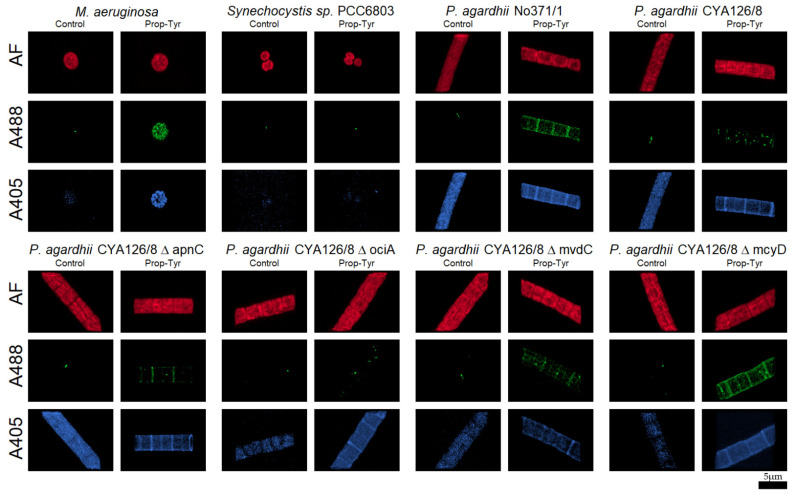
Confocal microscopy images from cells with labeled Prop-Tyr modified MC (*M. aeruginosa*) or AP (*P. agardhii* No371/1 and CYA126/8 and peptide knock out mutants). *Synechocystis* PCC6803 (lacking NRPS) was used as an additional control. Cells were grown in the presence of Prop-Tyr and labeled with ALEXA488 (A488) and ALEXA405 (A405), while AF indicates autofluorescence. The respective microscopic images for Phe-Az (Figure S2), Prop-Lys (Figure S3) and a comparison between all the treatments (Figure S4) are shown in the Supplementary Material.

Finally, total intensities were obtained and compiled for the three RGB channels, as well as the ratios between the different channels. Specifically, the green channel was used to measure the labeling with ALEXA488 and BODIPY 505/515, the blue channel measured the labeling effect with ALEXA405, while the red channel was used to measure the autofluorescence (AF) of the cells (Figure 2).

### 2.7. Colocalization Coefficient

Calculation of the colocalization coefficients between the differently labeled molecules was determined using Huygens Essential 20.04 software built in Huygens Colocalization Analyzer Advanced (Scientific Volume Imaging BV, Hilversum, The Netherlands). The background of the images was corrected using the integrated Costes method for background estimation, by calculating a regression line in which each point on the line is a combination of backgrounds in both channels and estimates the position where the Pearson coefficient of the background is zero [28].

Overall, the Pearson colocalization coefficients (PCC) measures the three dimensional voxel intensity covariance between the signal in two different channels [29]. PCC values range from −1 meaning a perfectly opposed distribution in the signal intensities and 1, which represents a complete overlap of the signals. The deconvolved images were analyzed using pairwise comparisons between the blue channel (ALEXA405) and the red channel (AF), thus calculating the PCC between the peptide intensity labeled with ALEXA405 and the natural AF. The intensities measured from the green channel (either BODIPY 505/515 or ALEXA488) were compared against the red channel (AF) for the colocalization between the lipids dyed with BODIPY 505/515 and the AF, or the intensity of ALEXA488 labeled peptides and AF. Finally, the blue channel (ALEXA405) and the green channel (BODIPY 505/515) were processed to determine the PCC between the labeled peptides and the labeled lipids.

## 3. Results

### 3.1. Growth Rate

In general, the supplementation of the medium using the non-natural amino acids Prop-Tyr and Prop-Lys did not reduce the growth rate of any of the strains, i.e., the growth rates varied between 0.27–0.45 d^−1^ for *Planktothrix* strains, while for cultures without non-natural amino acids the growth rates ranged between 0.32–0.47 d^−1^. In general, the unicellular cyanobacteria *M. aeruginosa* and *Synechocystis* PCC6803 showed higher growth rates ranging from 0.55–0.57 d^−1^ and 0.96–1.01 d^−1^ (Table S1).

In contrast to Prop-Tyr and Prop-Lys the addition of Phe-Az resulted in a decline in growth rate among all the strains. In particular, the growth rate of *Synechocystis* PCC6803 and *P. agardhii* CYA126/8 ΔapnC declined the most from Phe-Az addition, while *P. agardhii* CYA126/8 WT was less affected. Filaments from cultures grown in the presence of Phe-Az frequently showed a reduced AF, suggesting a reduced vitality of the cells.

Accordingly, obtained dry weights were smaller for cells harvested from cultures grown in the presence of Phe-Az when compared with Prop-Tyr or Prop-Lys treatments. Consequently, for Phe-Az relatively low amounts of biomass were available for peptide extraction (0.5–1.4 mg of dry weight) whereas for Prop-Tyr and Prop-Lys dry weights ranged from 0.9–3.7 mg and 1.5–4.0 mg, respectively.

### 3.2. Modified Peptides

As compared to control cells grown in the absence of non-natural amino acids we detected chemically modified MCs in *M. aeruginosa* peptide extracts carrying incorporated azide or alkyne moieties (Figure S5) Phe-Az, i.e., D-Asp-MC-Phe-azide [M+H]^+^ 1056.5 and MC-Phe-azide [M+H]^+^ 1070.5, or incorporating Prop-Lys, i.e., D-Asp-MC-Lys-alkyne [M+H]^+^ 1078.4 and MC-Lys-alkyne [M+H]^+^ 1092.5 or incorporating Prop-Tyr, i.e., D-Asp-

MC-Tyr-alkyne [M+H]^+^ 1069.5 and MC-Tyr-alkyne [M+H]^+^ 1083.5 were detected (Table 1). For all the modified variants, MS^n^ fragmentation revealed typical MC as the characteristic fragment of the Adda moiety [M+H]^+^ 135.0 or [M+H]^+^ 599.2 for Arg+Adda+Glu indicative of the conserved part of the MC molecule. Modified MC masses were predicted from the original MC molecular weight, subtracting the mass of the original AA substituted and adding the mass of the non-natural AA added (Table 1).

For *Synechocystis* PCC6803 the observed elution profile was not assigned to respective compounds, however, compared with *M. aeruginosa* no change in chromatogram elution profile was observed through the addition of non-natural AA (Figure S6). Modified AP variants carrying alkyne or azide moieties were detected in *P. agardhii* No371/1 carrying promiscuous ApnAA_1_ domains (Figure S7). Specifically, we were able to detect the incorporation of Phe-Az into the AP molecule presumably in its reduced form AP-Phe-azide [M+H]^+^ 843.3. In addition, modified AP-Tyr-alkyne [M+H]^+^ 882.4 and AP-Lys-alkyne [M+H]^+^ 891.5 were observed. Characteristic MS^n^ fragments included Lys+Val+Hty+H_2_O+H [M+H]^+^ 387.3, indicating an unmodified ring structure of the modified AP molecules.

*P. agardhii* CYA126/8 and its gene inactivation mutants for cyanopeptolin (ΔociA), microviridin (ΔmvdC) and MC (ΔmcyD) produced AP incorporating Phe-Az [M+H]^+^ 915.6 or Prop-Tyr [M+H]^+^ 954.6 and (Figure S8). Any AP structural variant was observed for the ΔapnC mutant (Figure S9). We were not able to detect AP incorporating Prop-Lys for CYA126/8 WT and its ΔociA, ΔmvdC, ΔmcyD peptide knock out mutants (Figures S10–S12). From AP 908 [M+H]^+^ 909.2 minus arginine (174.2) plus Prop-Lys (228.3) a theoretical mass [M+H]^+^ 963.3 was calculated which also was indicative of cyanopeptolin, i.e., [M+H]^+^ 863.2 (cyanopeptolin 880 -H_2_O), and [M+H]^+^ 961.1 (sulfated cyanopeptolin). Inspecting the ΔociA mutant revealed inactivated cyanopeptolin synthesis but no peak for EIC [M+H]^+^ 963.3 indicating a modified AP-Lys-alkyne structure.

#### 3.2.1. Peptide Labeling Intensity

Both ALEXA488 and ALEXA405 fluorophores resulted in peptide labeling either in *M. aeruginosa* or in *P. agardhii*. For all non-natural amino acids (Phe-Az, Prop-Lys and Prop-Tyr) labeling with ALEXA488 fluorophore was observed, however, labeling in the non-toxic cyanobacteria *Synechocystis* PCC6803 was not detected. In general, the green fluorophore ALEXA488 produced brighter signals with the highest intensities and a more pronounced difference from background fluorescence (Table 2). The blue fluorophore ALEXA405 also resulted in peptide labeling, however, the intensity was generally lower and the distinction from background fluorescence such as natural AF was reduced (Table 3).

The toxic cyanobacteria *M. aeruginosa* showed a significant increase in labeling intensities using all three amino acids when compared with control cells grown in the absence of any amino acid but treated under identical conditions. In particular, Phe-Az, Prop- Lys and Prop-Tyr resulted in five- and four-fold increases in the average intensity compared to the control (Table 2). Correspondingly, strong labeling was detected for Prop-Lys and Prop-Tyr fed cultures when ALEXA405 was applied, but the increase was only 1–2.4 fold. Since ALEXA405 was only available as an azide it could not be applied for MC or AP labeling carrying the Phe-Az moiety (Table 3). In contrast, *Synechocystis* PCC6803 did not show any intensity increase with any of the amino acids fed and the application of ALEXA405.

Among the *Planktothrix* strains significant increase in intensity using the green ALEXA488-azide was consistently detected for No371/1. For Phe-Az, however, fluorescence intensity was not significantly increased using strain No371/1 (0.6 ± 0.7 vs. 1.3 ± 0.6, without and with ALEXA488-alkyne respectively). Results were less consistent using the blue fluorophore ALEXA405: For strain No371/1 only Prop-Lys resulted in a significant increase of fluorescence intensity (1.2 ± 0.2 vs. 0.8 ± 0.2 with and without ALEXA405-azide respectively, *p* < 0.001), while no evidence of labeling was found in the cultures fed with Prop-Tyr (0.9 ± 0.3 vs. 1.0 ± 0.2).

For CYA126/8 WT, Phe-Az significantly increased green fluorescence intensity, i.e., 2.0 ± 0.5 vs. 1.2 ± 0.2, with and without ALEXA488-alkyne respectively while an increase in intensity using Prop-Lys and Prop-Tyr was less pronounced (Table 2). Labeling with ALEXA405-azide produced an increased intensity with Prop-Lys and Prop-Tyr, suggesting significant labeling of filaments (2.3 ± 0.5 vs. 1.6 ± 0.3 and 0.9 ± 0.2 vs. 1.0 ± 0.2, with and without ALEXA405-azide respectively, *p* < 0.001). In comparison with CYA126/8 WT, labeling intensity was found to be less reduced in the AP synthesis mutant ΔapnC, i.e., a slightly increased intensity for Prop-Tyr was observed using ALEXA488 (1.3 ± 0.4 vs. 0.7 ± 0.2, *p* < 0.001) and ALEXA405 (1.4 ± 0.2 vs. 0.6 ± 0.1, *p* < 0.05) while no increase was detected for the Prop-Lys fed cultures labeled with ALEXA405.

The cyanopeptolin synthesis mutant CYA126/8 ΔociA showed significantly increased green fluorescence intensity for all the non-natural amino acids supplements. In comparison with the CYA126/8 WT the ΔociA mutant showed higher fluorescence intensity, i.e., 1.3–2.0 fold (CYA 126/8 WT) vs. 1.8–2.4 fold (ΔociA mutant) for all three amino acids. Using ALEXA405 blue fluorescence intensity only increased under Prop-Tyr feeding conditions (1.6 ± 0.3 vs. 1.0 ± 0.3, *p* < 0.05).

Similar results were obtained for microviridin (ΔmvdC) and MC (ΔmcyD) gene knock out mutants both showing increased intensity of green fluorescence using ALEXA488, i.e., 1.7-fold vs. 0.6–0.9 fold for Prop-Lys and 1.5–1.6-fold vs. 0.7–0.9-fold for Prop-Tyr. Significant ALEXA488 labeling using Phe-Az was observed for the ΔmvdC mutant only. ALEXA405 labeling again was overall reduced and was found increased for the ΔmvdC and the ΔmcyD strain fed with Prop-Lys (Table 3).

#### 3.2.2. Peptide Intensity/Autofluorescence Ratio

**Figure 3:**
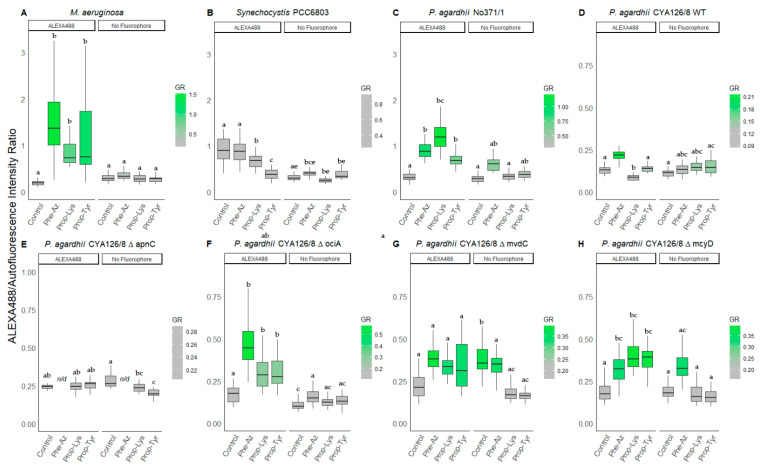
The ratio of green fluorescence intensity (ALEXA488) to red intensity (AF) for individual cells or filaments from (**A**) *M. aeruginosa*, (**B**) *Synechocystis* PCC6803, and (**C**) *P. agardhii* strain No371/1, (**D**) CYA126/8 WT, and (**E**–**H**) CYA126/8 gene knock out mutants: (**E**) ΔapnC (inactivated AP synthesis), (**F**) ΔociA (inactivated cyanopeptolin synthesis), (**G**) ΔmvdC (inactivated microviridin synthesis), and (**H**) ΔmcyD (inactivated MC synthesis) using non-natural amino acids (Phe-Az, Prop-Lys, and Prop-Tyr). Controls were grown without amino acid addition but used for the chemical reaction under identical conditions. No Fluorophore indicates filaments or cells grown with amino acid addition but no subsequent labeling by click-chemical reaction. The gradient in coloring was defined for each strain separately using the average intensity from the control cultures. Superscripts indicate statistically significant different subgroups after overall difference was found (*p* < 0.05).

Both genera *M. aeruginosa* and *P. agardhii* increased in the ratio between ALEXA488 and AF with either of the three non-natural amino acids. Whereas *Synechocystis* PCC6803 did not show this effect. According to the increased green intensity (Table 2), the highest signal ratios were observed for *M. aeruginosa*, i.e., a median ratio of 1.5 for Phe-Az, 0.9 for Prop-Lys, and 1.2 for Prop-Tyr as compared with 0.2 for controls (Figure 3).

Among *Planktothrix* strains, *P. agardhii* No371/1 produced the highest ratios, i.e., 1.0 for Phe-Az, 1.2 for Prop-Lys, and 0.7 for Prop-Tyr when compared with 0.3 for controls. For CYA126/8 WT the ratios using the feeding treatments Prop-Lys and Prop-Tyr remained unaltered, while Phe-Az (0.2) increased when compared with controls (0.1). The ΔapnC mutant did not show increased ratios of peptide intensity vs. AF between the treatments.

**Figure 4:**
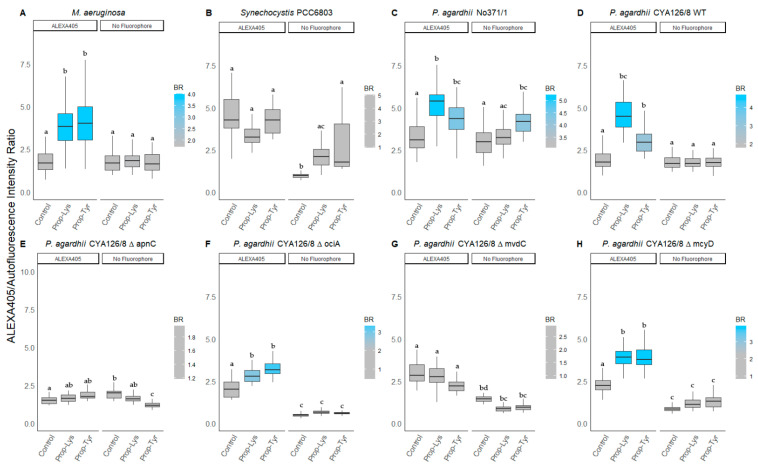
The ratio of blue fluorescence intensity (ALEXA405) to red intensity (AF) for individual cells or filaments from (**A**) *M. aeruginosa*, (**B**) *Synechocystis* PCC6803, and (**C**) *P. agardhii* strain No371/1, (**D**) CYA126/8 WT, and (**E**–**H**) CYA126/8 gene knock out mutants: (**E**) ΔapnC (inactivated AP synthesis), (**F**) ΔociA (inactivated cyanopeptolin synthesis), (**G**) ΔmvdC (inactivated microviridin synthesis), and (**H**) ΔmcyD (inactivated MC synthesis) using non-natural amino acids (Phe-Az, Prop-Lys, and Prop-Tyr). Controls were grown without amino acid addition but used for the chemical reaction under identical conditions. No Fluorophore indicates filaments or cells grown with amino acid addition but no subsequent labeling by click-chemical reaction. The gradient in coloring was defined for each strain separately using the average intensity from the control cultures. Superscripts indicate statistically significant different subgroups after overall difference was found (*p* < 0.05).

The mutants ΔociA, ΔmvdC and ΔmcyD showed rather similar response, i.e., ratios increased for Phe-Az (0.3–0.6), Prop-Lys (0.3–0.4), Prop-Tyr (0.3–0.4) vs. 0.2 for controls. ALEXA405 ratios were calculated by dividing the blue labeling intensity by AF (Figure 4). In general, both ALEXA488 and ALEXA405 signal ratios showed comparable increases, whereas *Synechocystis* PCC6803 did not show response compared to the control.

Similarly, ALEXA405 labeling (Table 3) also resulted in significantly increased ratios for *M. aeruginosa*, i.e., median ratio 4.0 for Prop-Lys, 4.0 for Prop-Tyr vs. 1.8 for controls. For *P. agardhii* strain No371/1 the ratio was 5.2 for Prop-Lys, 4.3 for Prop-Tyr vs. 3.3 for control. Among *P. agardhii* CYA126/8 WT the median ratio ranged between 4.7 (Prop-Lys), 3.0 (Prop-Tyr) to 2.0 (control). Using ALEXA405 labeling, the ΔapnC mutant did not reveal a change in signal ratio, i.e., the median ratio varied from 1.7 (Prop-Lys), 1.9 (Prop-Tyr), and 1.7 (Control). In contrast to ΔapnC, the mutants ΔociA and ΔmcyD showed a similar response, i.e., a ratio of 2.9–3.9 (Prop-Lys), 3.3–3.9 (Prop-Tyr) vs. 2.0–2.3 (controls). For unknown reasons, no significant increase in ratio was detected when testing Prop-Lys and Prop-Tyr for the microviridin mutant strain ΔmvdC.

### 3.3. Lipids

#### 3.3.1. BODIPY Labeling

In general, BODIPY 505/515 signals were visible among unicellular genera *Microcystis* and *Synechocystis* as well as the filamentous genus *Planktothrix* (Table 4). For *P. agardhii* the results were variable, i.e., No371/1 showed increased labeling intensities, while CYA126/8 WT did not. On the other hand, CYA126/8 mutants ΔociA and ΔmcyD showed increased green fluorescence intensity irrespective of the feeding treatment (Figures S13–S16).

Overall, *Synechocystis* PCC6803 and *M. aeruginosa* showed a pronounced increase in the BODIPY 505/515 signal, i.e., 1.9–3.0-fold for *M. aeruginosa* and 1.3–5.2 fold for *Synechocystis* PCC6803 (Table 4). In comparison, the intensity of lipid staining of *P. agardhii* was moderate only, i.e., 1.2–1.7 fold for No371/1 and undetectable for CYA126/8 WT. The CYA126/8 mutants ΔapnC also did not reveal much BODIPY induced fluorescence. In contrast mutants ΔociA, ΔmcyD and partly ΔmvdC showed a distinct response in green fluorescence intensity, i.e., 2–2.6-fold (ΔociA), 1.3–2.7 fold (ΔmvdC), 0.9–3.5-fold (ΔmcyD).

#### 3.3.2. BODIPY/Autofluorescence Ratio

**Figure 5:**
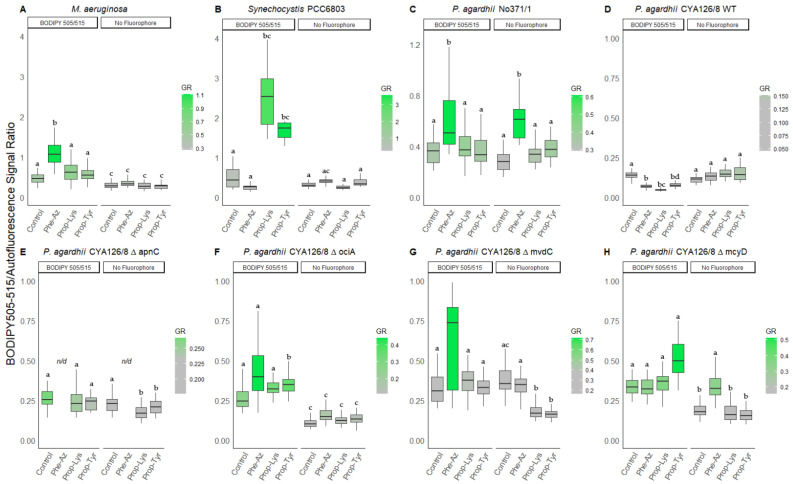
Ratio of green fluorescence intensity (BODIPY) and red intensity (AF) for individual cells or filaments from (**A**) *M. aeruginosa*, (**B**) *Synechocystis* PCC6803, and (**C**) *P. agardhii* strain No371/1, (**D**) CYA126/8 WT, and (**E**–**H**) CYA126/8 gene knock out mutants: (**E**) ΔapnC (inactivated AP synthesis), (**F**) ΔociA (inactivated cyanopeptolin synthesis), (**G**) ΔmvdC (inactivated microviridin synthesis), (**H**) ΔmcyD (inactivated MC synthesis) using non-natural amino acids (Phe-Az, Prop-Lys, Prop-Tyr). Controls were grown without amino acid addition. No Fluorophore indicates filaments or cells grown with amino acid addition but no BODIPY labeling. The gradient in coloring was defined using the average intensity from the control cultures without supplementing amino acids. Superscripts indicate statistically significant different subgroups after overall difference was found (*p* < 0.05); n/d: no data.

According to lipid signal intensities (Table 4) unicellular cyanobacteria *M. aeruginosa* and *Synechocystis* PCC6803 showed the highest intensity ratios, while *Planktothrix* strains only showed a moderate response (Figure 5). For *M. aeruginosa* the BODIPY median ratio ranged from 0.5–1.1 vs. 0.3–0.4 (controls). For *Synechocystis* PCC6803 the same median ratio ranged from 0.50–3.6 (Phe-Az not included) vs. 0.3–0.4 (controls).

In contrast, among *P. agardhii* strains, No371/1 the BODIPY signal was rather low, i.e., 0.4–0.6 vs. 0.3–0.6 in controls. After any increase in fluorescence intensity, the median ratio for CYA126/8 WT also did not differ from treatments without labeling, i.e., 0.1–0.1 vs. 0.1–0.2 (controls). Similarly, for the ΔapnC mutant, no significant change in green intensity was observed. In contrast, median ratios increased significantly for mutants ΔociA (0.3–0.4 vs. 0.1–0.2), ΔmvdC (0.3–0.7 vs. 0.2–0.4), ΔmcyD (0.3–0.5 vs. 0.2–0.3).

### 3.4. Colocalization of Peptides vs. Autofluorescence and Lipids

**Figure 6:**
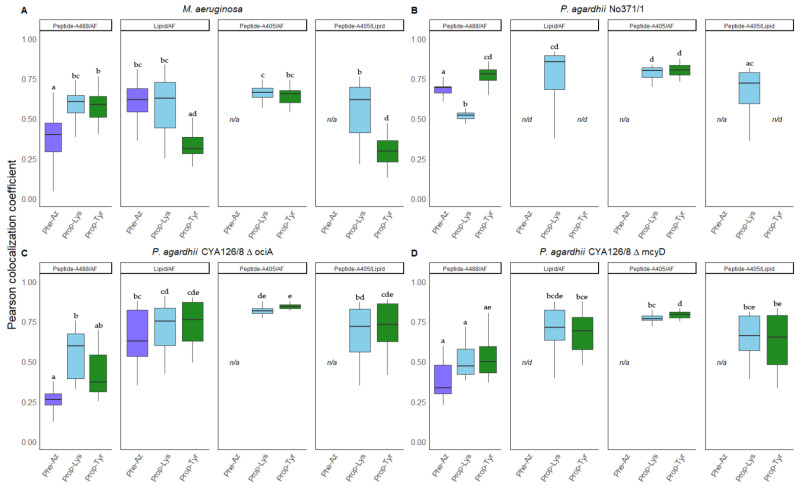
Pearson colocalization coefficients (PCC) calculated for (**A**) *M. aeruginosa*, (**B**) *P. agardhii* No371/1, (**C**) *P. agardhii*, ΔociA and (**D**) *P. agardhii*, ΔmcyD strains showing both significant labeling with ALEXA488 (green channel), ALEXA405 (blue channel) and BODIPY 505/515 (green channel). Pairwise statistical analysis between the PCC values were calculated i.e., ALEXA488 labeled peptides and natural autofluorescence (AF), BODIPY 505/515 stained lipids and AF, ALEXA405 labeled peptides and natural AF and ALEXA405 labeled peptides, and BODIPY 505/515 stained lipids. Superscripts indicate statistically significant different subgroups after overall difference was found (*p* < 0.05); n/d: no data; n/a: not applicable.

From the same deconvolved images used for comparing signal intensities, the Pearson colocalization coefficient (PCC) was calculated pairwise between blue, green, and red fluorescence signals (Figure 6). The ALEXA405 labeled peptide signal disposition relative to the AF and lipids were measured with the voxel comparison of the total intensity in the blue channel (A405) against the red (AF) and green (BODIPY 505/515) channels. The same procedure was followed for determining the lipid (green channel) colocalization coefficient against the AF (red channel) and the ALEXA488 labeled peptide distribution (green channel) against the AF (red channel). Since significant BODIPY/peptide labeling only occurred in *M. aeruginosa*, *P. agardhii* No371/1, CYA126/8 ΔociA and CYA126/8 ΔmcyD only these strains were used for colocalization analysis.

In general, ALEXA488 labeled peptides showed relatively low spatial correlation with AF i.e., for *M. aeruginosa* PCC values varied between 0.55 ± 0.15, for *P. agardhii* No371/1 between 0.68 ± 0.12, and for CYA126/8 mutants ΔociA and ΔmcyD between 0.36 ± 0.17 and 0.47 ± 0.13, respectively.

For lipids, the PCC values with the AF were often more variable and in general increased i.e., *M. aeruginosa* PCC values of 0.52 ± 0.18 were found, for *P. agardhii* No371/1 0.86 ± 0.15, and for CYA126/8 mutants ΔociA 0.70 ± 0.15 and ΔmcyD 0.70 ± 0.12. Thus, regarding autofluorescence (AF) both ALEXA488 labeled peptides and BODIPY stained lipids showed a more heterogeneous distribution in the cell. When compared with ALEXA488 for most treatments, the PCC between lipids and AF was found to have significantly increased.

In contrast to ALEXA488 labeled peptides, the ALEXA405 labeled peptides showed higher and less variable correlation with AF, i.e., for *M. aeruginosa* PCC values ranged from 0.65 ± 0.05, for No371/1 0.80 ± 0.04, for ΔociA 0.83 ± 0.03 and for ΔmcyD 0.78 ± 0.03. Furthermore, the correlation between ALEXA405 labeled peptides and BODIPY 505/515 stained lipids was found to be overall variable and reduced, i.e., for *M. aeruginosa* calculated values were 0.44 ± 0.19, for No371/1 between 0.72 ± 0.17, and for CYA126/8 mutants ΔociA and ΔmcyD PCC values ranged between 0.72 ± 0.14 and 0.65 ± 0.15 respectively. In summary, when compared with AF, the spatial correlation between ALEXA405 labeled peptides and lipids was found rather reduced than increased.

## 4. Discussions

In general, the supplementation of the non-natural amino acids Prop-Lys and Prop-Tyr did not produce a detectable adverse effect in the growth of the strains in this study. In contrast, Phe-Az feeding resulted in a decrease in the growth rate for many strains (Table S1). The inhibitory effects of sodium azide (NaN_3_) are well documented [34,35,36]. Sodium azide inhibits the cytochrome oxidase in bacteria. Similar to NaN_3_ the azide ion from Phe-Az may block the catalytic domain of cytochrome-c-oxidase binding oxygen.

When comparing LC-MS elution profiles (Figures S5–S12), a rather specific incorporation of the non-natural amino acids into the MCs or APs produced by NRPS synthesis has been observed, i.e., using strains carrying promiscuous A-domains such as McyBA1 of *M. aeruginosa* and *Planktothrix agardhii* ApnAA_1_ in strains No371/1 and CYA126/8 [2]. Thereby, newly modified MC/AP structures, presumably carrying the Phe-Az moiety either in pos.2 of the MC molecule or in the exocyclic position of the AP molecule were obtained. In addition, no labeling in the non-toxic strain *Synechocystis* PCC6803 was detected, demonstrating that unspecific incorporation of non-natural amino acids Phe-Az, Prop-Lys and Prop-Tyr into anabolic pathways other than NRPS did not occur. Nevertheless, the ΔapnC mutant revealed Prop-Tyr labeling, possibly suggesting ongoing partial AP synthesis. The AP synthesis pathway through NRPS is starting from ApnAA1 with activating and condensing the first exocyclic amino acid to the conserved lysine via the characteristic ureido-linkage [4]. Thus even in ΔapnC mutant, the AP synthesis may continue with a non-natural AA through the presumably intact NRPS ApnA2 and ApnB [4], and a linear AP peptide fragment comprising the three amino acids Tyr/Arg-Lys-Val might still be synthesized.

Currently the observed, rather moderate, Prop-Tyr labeling for the *P. agardhii* ΔapnC mutant strain does not support our hypothesis on Prop-Tyr incorporation during MC biosynthesis, i.e., identification of its derivation from D-Asp-MC-Tyr-alkyne [M + H] 1069.5 could not be unequivocally performed. However, since neither the ALEXA488 nor the ALEXA405 fluorescence to AF ratios were affected, we conclude that the signal increase from Tyr-alkyne in the ΔapnC mutant strain was generally minor.

Nonetheless, the incorporation of Prop-Lys could not be shown for AP synthesis using strain CYA126/8 WT or its mutants ΔociA, ΔmvdC, ΔmcyD. It is possible, that low proportion of putative AP incorporating Prop-Lys is coeluting with natural AP 915, which occur with more than 50% proportion of total APs in strain CYA126/8. Since cyanobacteria are in general well known for their ability to uptake various AA from the medium [37], the labeling of non-natural AA transported across the inner cell membrane potentially creates artificial results. In this study for nearly all strains including *Synechocystis* PCC6803 low-albeit-significant amounts of non-natural AA (Phe-Az, Prop-Lys and Prop-Tyr) were detected during inspection of LC-MS chromatograms (Figures S5–S12). Since *Synechocystis* PCC6803 also contained non-natural AA (Figure S6) but did not show increase in ALEXA488 or ALEXA405 signal intensity (Figure 3 and Figure 4), this potential artifact is considered less likely.

Using the ALEXA488 fluorophore the CYA126/8 WT strain showed overall lower intensity when compared to the peptide knock-out mutants ΔociA, ΔmvdC, ΔmcyD as well as strain No371/1 (Figure 3 and Figure 4). It has been suggested earlier that metabolite synthesis between different NRPS pathways might interfere and might depend on the availability of building blocks, precursors, and other resources. For example, the feeding of the putatively limiting amino acid D-L-homotyrosine led to the increased production and subsequent structural elucidation of AP 908 and 915 vs. cyanopeptolin 880 and 960 for strain CYA126/8 WT [33]. Thus, for unknown reasons the inactivation of cyanopeptolin, microviridin, or MC synthesis might favor the integration of unnatural AA into AP exocyclic position No1. Furthermore, the ApnAA1 domain of strain No371/1 has been found more promiscuous when compared with strain CYA126/8 WT [2], i.e., AP C carrying lysine in pos.1 is produced in low amounts by strain No371/1 but not by strain CYA126/8. Thus, strain No371/1 might be more efficient in the integration of the three tested non-natural AA into the exocyclic position of the AP molecule (Phe-Az, Prop-Lys Prop-Tyr).

Labeling with the blue fluorophore ALEXA405-azide yielded acceptable fluorescence signals for *M. aeruginosa* but lower signals for *P. agardhii* when grown in the presence of Prop-Lys and Prop-Tyr. In particular, peptide labeling results in *P. agardhii* CYA126/8 WT, and its peptide knock out mutants were more variable when compared to the data obtained with ALEXA488. One reason for the higher variability might be the optical interference of ALEXA405 with photosynthetic pigments. Indeed, AF recorded at 405 nm was found much higher than AF recorded at 490 nm (Figure 2 vs. Figures S2–S4). Consequently, an increased background fluorescence for *P. agardhii* was found in the blue range for all three non-natural AA treatments, which possibly interferes with peptide labeling using ALEXA405 (Figure 3 vs. Figure 4). In general, the genus *Planktothrix* has shown higher AF when compared to *Microcystis* and *Synechocystis*, demonstrating an important ecophysiological trait in response to the metalimnetic and shade (low light)-adapted lifeform of *Planktothrix* in general. Technically, ALEXA405 was found useful for *M. aeruginosa* and the co-labeling of peptides vs. other intracellular organelles using green fluorescent dyes. However, for *Planktothrix* the stronger autofluorescence will possibly limit the applicability of ALEXA405 to strains with naturally lower AF.

Nevertheless, as a proof of concept, we differentially labeled lipids in parallel to labeled modified MC vs. AP variants and compared lipid labeling intensity between strains (Figure 5). Both unicellular cyanobacteria revealed significant BODIPY labeling suggesting that our modified BODIPY protocol was functional. For *Planktothrix*, however, lipid labeling results appeared to be strain specific. While CYA126/8 WT strain and ΔapnC did not show any significant BODIPY signal, the three other mutants (ΔociA, ΔmvdC, ΔmcyD) showed higher intensity. Out of the three mutants showing increased BODIPY intensity, the ΔmvdC showed a decreased BODIPY/AF ratio, probably because of the increased AF.

Finally, only four strains were retained to quantify and compare the intracellular location of the peptides using the PCC between the ALEXA405 labeled peptide and the BODIPY 505/515 lipid signal. The distribution of modified peptides labeling signals with either ALEXA405 (blue channel) or ALEXA488 (green channel) against AF was also compared. As a result, compared with the correlation between the ALEXA405 (blue channel) and AF, the PCC values between the modified peptides (blue channel) and lipids (green channel) were found in a comparable upper range or decreasing. These results suggest that for *M. aeruginosa* and *P. agardhii* No371/1 both strains showed a relatively high spatial correlation between labeled peptides with both fluorophores against the natural AF (Figure 6). Previous studies using cryosectioning also detected MCs alongside the thylakoidal membranes [11,38]. Other studies reported the majority of MCs alongside polyphosphate bodies [39].

On the other hand, *P. agarhii* CYA126/8 mutants ΔociA and ΔmcyD showed an overall lower correlation between peptide ALEXA488 with AF but a similar range in PCC between lipids and AF distribution (Figure 6). Indeed, from images, a more distinct pattern of labeled modified APs with ALEXA488 and ALEXA405, seemingly locating APs close to the septa separating different cells of the filament has been recognized. Whereas detection of modified APs alongside cellular septa is more evident for ALEXA488 (Figure 2), labeled peptides with ALEXA405 did not produce a reduction in PCC values probably due to the higher interference by AF (Figures S2–S4). Nonetheless, our results suggest that modified APs in *P. agardhii* ΔociA and ΔmcyD may present a different intracellular disposition from *M. aeruginosa* or *P. agardhii* No371/1. It might be worthwhile to address the reproducibility of the intracellular location of APs in *Planktothrix* strains using the ALEXA488 labeling technique.

The protocols here described offer us a new tool to investigate the distribution of modified peptides inside the cell. More advanced image analysis, including 3D rendering would allow acquisition of additional measures such as volume of the detected modified MCs/APs, the number of individual vesicles, or distance from the membranes.

New developments and description of proteins associated with filamentous cyanobacteria, as the description of the cyanobacterial cell division protein CyDiv [40] may allow us to confirm the potential location of the labeled peptides alongside the cellular septa. This test would consist of the simultaneous application of the current CuAAC labeling technique and suitable staining of CyDiv protein followed by colocalization coefficient analysis.

## 5. Conclusions

In summary, there is little evidence that other nontarget peptides than APs or MCs are modified. Together with the positive labeling results for *M. aeruginosa* and the negative labeling results from *Synechocystis* PCC6803 and the CYA126/8 AP mutant we have reason to state that labeling is indeed related to AP/MC to a significant amount (and less to nontarget compounds). The generally increased AP labeling in the CYA126/8 mutants ΔociA, ΔmvdC, ΔmcyD as compared to the WT can be because more resources are available for peptide synthesis since one peptide synthesis pathway is inactive. The results do not support the role of lipids spatially related to the intracellular accumulation of (chemically modified) MCs vs. APs. In other words, lipids were found less correlated with the MC/AP signal than the MC/AP signal with AF.

## Figures and Tables

**Table 1:** Protonated masses [M+H]^+^ of modified MCs and APs structural variants for the cyanobacteria *M. aeruginosa* and *P. agardhii*. Masses of the modified peptides were calculated by replacing the natural amino acid with the non-natural amino acid at pos.2 (MC) or pos.1 (AP). Observed MS^n^ fragments are listed according to [30,31] for MCs or [2,32,33] for APs. Non-natural amino acid incorporation is calculated as a percentage of natural structural variants produced under control conditions.

Peptide	Original Mass [M+H]^+^	Exchange AA	Non-Natural AA	Predicted Mass [M+H]^+^	Detected Mass [M+H]^+^	Fragmentation MS^n^ [M+H]^+^	Proportion of Modified MC or AP (%)	Elution Time (min)
*M. aeruginosa*								
D-Asp-MC-YR	1031.5	181.4 (Tyr)	206.1 (Phe-Az)	1056.4	1056.5	nd ^1^	1	38.1
MC-YR	1045.5	“	“	1070.4	1070.5	599.3 ^2^	2	38.5
D-Asp-MC-YR	1031.5	“	228.3 (Prop-Lys)	1078.5	1078.4	nd ^1^	<1	32.6
MC-YR	1045.5	“	“	1092.6	1092.5	599.3 ^2^	2	33.7
D-Asp-MC-YR	1031.5	“	219.2 (Prop-Tyr)	1069.4	1069.5	599.3 ^2^, 135.0 ^3^	2	37.4
MC-YR	1045.5	“	“	1083.5	1083.5	949.4 ^4^, 599.3 ^2^, 135.0 ^3^	45	38.1
*P. agardhii* No371/1								
AP-A	844.4	181.4 (Tyr)	206.1 (Phe-Az)	869.3 (843.3 reduced)	843.3	637.4 ^5^, 619.4 ^6^, 534.4 ^7^, 460.3 ^8^, 387.3 ^9^	14	16.7
“	“	“	228.3 (Prop-Lys)	891.5	891.5	637.4 ^5^, 619.4 ^6^, 534.4 ^7^, 460.3 ^8^, 387.3 ^9^	81	25.1
“	“	“	219.2 (Prop-Tyr)	882.4	882.4	nd ^1^	4	31.9
*P. agardhii* CYA126/8 WT and ΔociA, ΔmvdC, ΔmcyD mutants								
AP-908	909.2	174.2 (Arg)	206.1 (Phe-Az)	941.1 (915.1 reduced)	915.1	709.2 ^10^, 691.2 ^11^, 387.2 ^9^, 500.2 ^12^, 277.1 ^13^	3–5	18.5
AP-915	916.2	181.4 (Tyr)	219.2 (Prop-Tyr)	954.2	954.2	709.2 ^10^, 691.2 ^11^, 387.1 ^9^, 500.1 ^12^, 277.1 ^13^	3	33.5

^1^: no data; ^2^: Arg + Adda + Glu + H; ^3^: Adda side chain; ^4^: MC-YR minus Adda side chain + H; ^5^: Lys + Val + Hty + MAla + Phe + 2H; ^6^: Lys + Val + Hty + H_2_O + MAla + Phe + H; ^7^: Lys + Val + Hty + H_2_O + Phe + H; ^8^: Lys + Val + MAla + Phe + 2H; ^9^: Lys + Val + Hty + H_2_O + H; ^10^: Lys + Val + Hty + MHty + Ile + 2H; ^11^: Lys + Val + Hty + H_2_O + MHty + Ile + H; ^12^: Lys + Val + MHty + H_2_O + Ile + H; ^13^: Hty + Val + H.

**Table 2:** Average (±SD) min–max green fluorescence intensity obtained for individual treatments using non-natural amino acid feeding (Phe-Az, Prop-Lys, and Prop-Tyr) and subsequent labeling by ALEXA488 using copper-catalyzed azid-alkyne cycloaddition (CuAAC). The intensity was divided by the average intensity of control filaments or cells, i.e., cells which were grown without amino acid addition but used for the chemical reaction under identical conditions. No Fluorophore indicates filaments or cells grown with amino acid addition but no subsequent labeling by the click-chemical reaction. n: number of individual filaments (*Planktothrix*) or cells (*Microcystis*, *Synechocystis*).

		ALEXA488 ^1^			No Fluorophore ^1^		
	n	Phe-Az	Prop-Lys	Prop-Tyr	Phe-Az	Prop-Lys	Prop-Tyr
*M. aeruginosa* Hofbauer	43	4.6 ± 2.2 ^a^	4.4 ± 3.7 ^a^	5.3 ± 2.6 ^a^	1.2 ± 0.3 ^b^	1.0 ± 0.3 ^b^	1.0 ± 0.3 ^b^
		1.1–12.5	1.6–19.4	1.1–13.0	0.6–1.7	0.5–1.6	0.5–1.6
*Synechocystis* PCC6803	20	1.0 ± 0.8 ^ab^	0.5 ± 0.6 ^a^	0.3 ± 0.1 ^a^	1.5 ± 0.6 ^b^	2.1 ± 0.8 ^bc^	2.4 ± 1.8 ^bc^
		0.3–3.2	0.2–2.7	0.1–0.4	0.8–2.6	0.8–4.0	0.9–6.9
*P. agardhii* No371/1	16	1.3 ± 0.6 ^a^	4.0 ± 1.2 ^b^	1.9 ± 0.4 ^c^	0.6 ± 0.7 ^a^	0.8 ± 0.2 ^a^	0.9 ± 0.2 ^a^
		0.5–3.3	2.1–7.5	1.3–2.7	0.1–2.2	0.5–1.2	0.6–1.2
*P. agardhii* CYA126/8 WT	38	2.0 ± 0.5 ^b^	1.3 ± 0.3 ^ac^	1.6 ± 0.2 ^ad^	1.2 ± 0.2 ^a^	1.2 ± 0.3 ^a^	1.4 ± 0.5 ^a^
		0.5–2.5	0.8–1.9	0.8–1.9	0.8–1.7	0.8–1.7	0.8–2.3
*P. agardhii* CYA126/8 ΔapnC	20	n/d	1.0 ± 0.1^a^	1.3 ± 0.4 ^b^	n/d	0.9 ± 0.2 ^ac^	0.7 ± 0.2 ^c^
			0.8–1.3	1.0–2.6		0.7–1.3	0.5–1.1
*P. agardhii* CYA126/8 ΔociA	38	1.8 ± 0.5 ^a^	2.5 ± 0.7 ^a^	2.4 ± 0.6 ^a^	1.0 ± 0.2 ^b^	1.1 ± 0.5 ^b^	1.0 ± 0.3 ^b^
		1.0–3.1	1.5–3.6	1.4–3.6	0.6–1.3	0.6–2.5	0.2–2.0
*P. agardhii* CYA126/8 ΔmvdC	40	0.9 ± 0.3 ^a^	1.7 ± 0.3 ^ab^	1.5 ± 0.7 ^a^	0.6 ± 0.2 ^c^	0.6 ± 0.2 ^c^	0.7 ± 0.1 ^c^
		0.6–1.9	1.2–2.4	0.6–2.5	0.3–0.9	0.4–1.1	0.5–0.8
*P. agardhii* CYA126/8 ΔmcyD	39	0.9 ± 0.3 ^b^	1.7 ± 0.3 ^a^	1.6 ± 0.3 ^a^	1.0 ± 0.3 ^b^	0.9 ± 0.3 ^b^	0.9 ± 0.2 ^b^
		0.5–1.9	1.3–2.3	1.0–2.2	0.6–1.7	0.5–1.5	0.6–1.5

^1^ For each strain, treatments were compared using Kruskal–Wallis ANOVA on Ranks. We found statistically significant differences between the treatments (*p* < 0.001) in all of them. Superscripts indicate homogeneous subgroups not significantly different at *p* < 0.05 using post-hoc pairwise comparison (Tukeyߣs test); n/d: no data.

**Table 3:** Average (±SD) min–max blue fluorescence intensity obtained for individual treatments using non-natural amino acid feeding (Prop-Lys and Prop-Tyr) and subsequent labeling by ALEXA405 using copper-catalyzed azide-alkyne cycloaddition (CuAAC). The intensity was divided by the average intensity of control filaments or cells, i.e., cells were grown without amino acid addition but used for the chemical reaction under identical conditions. No Fluorophore indicates filaments or cells grown with amino acid addition but no subsequent labeling by the click-chemical reaction. n: number of individual filaments (*Planktothrix*) or cells (*Microcystis, Synechocystis*).

		ALEXA405 ^1^		No Fluorophore ^1^	
	(n)	Prop-Lys	Prop-Tyr	Prop-Lys	Prop-Tyr
*M. aeruginosa* Hofbauer	50	1.9 ± 0.6 ^a^	2.4 ± 0.9 ^a^	1.1 ± 0.4 ^bc^	1.0 ± 0.4 ^b^
		0.8–3.5	0.5–3.7	0.4–2.1	0.3–1.9
*Synechocystis* PCC6803	14	0.9 ± 0.6 ^a^	0.9 ± 0.3 ^a^	5.5 ± 2.4 ^b^	5.6 ± 5.3 ^b^
		0.3–2.1	0.4–1.5	2.1–9.7	1.6–19.8
*P. agardhii* No371/1	39	1.2 ± 0.2 ^a^	0.9 ± 0.3 ^a^	0.8 ± 0.2 ^ab^	1.0 ± 0.2 ^a^
		0.7–1.7	0.4–1.5	0.5–1.1	0.6–1.4
*P. agardhii* CYA126/8 WT	38	2.3 ± 0.5 ^c^	1.6 ± 0.3 ^a^	0.9 ± 0.2 ^b^	1.0 ± 0.2 ^b^
		1.3–3.6	0.9–2.2	0.6–1.5	0.7–1.5
*P. agardhii* CYA126/8 ΔapnC	20	1.0 ± 0.2 ^a^	1.4 ± 0.2 ^b^	0.9 ± 0.1 ^a^	0.6 ± 0.1 ^d^
		0.7–1.3	1.0–1.9	0.8–1.1	0.5–0.8
*P. agardhii* CYA126/8 ΔociA	39	1.4 ± 0.3 ^ac^	1.6 ± 0.3 ^a^	0.7 ± 0.2 ^bc^	1.0 ± 0.3 ^b^
		0.9–2.0	1.2–2.4	0.7–2.2	0.4–1.7
*P. agardhii* CYA126/8 ΔmvdC	40	1.1 ± 0.3 ^a^	1.0 ± 0.2 ^a^	0.8 ± 0.2 ^c^	1.0 ± 0.2 ^a^
		0.6–1.7	0.6–1.4	0.5–1.3	0.8–1.3
*P. agardhii* CYA126/8 ΔmcyD	38	1.5 ± 0.2 ^a^	1.3 ± 0.3 ^a^	1.2 ± 0.3 ^abd^	1.5 ± 0.3 ^ab^
		1.0–1.9	0.8–1.8	0.9–2.0	0.8–2.2

^1^ For each strain, treatments were compared using Kruskal–Wallis ANOVA on Ranks. We found statistically significant differences between the treatments (*p* < 0.001) in all of them. Superscripts indicate homogeneous subgroups not significantly different at *p* < 0.05 using post-hoc pairwise comparison (Tukeyߣs test).

**Table 4:** Average (± SD) min–max green fluorescence intensity obtained for individual treatments using non-natural amino acid feeding (Phe-Az, Prop-Lys, Prop-Tyr) and subsequent labeling by BODIPY. The intensity was divided by the average intensity of control filaments or cells without BODIPY incubation (No Fluorophore). *n*: number of individual filaments (*Planktothrix*) or cells (*Microcystis*, *Synechocystis*).

		BODIPY 505/515				No Fluorophore			
	(n)	Phe-Az	Prop-Lys	Prop-Tyr	Control	Phe-Az	Prop-Lys	Prop-Tyr	Control
*M. aeruginosa* Hofbauer	50	2.9 ± 0.6 ^b^	2.0 ± 0.8 ^a^	3.0 ± 1.1 ^b^	1.9 ± 1.0 ^a^	1.0 ± 0.3 ^c^	1.0 ± 0.5 ^c^	1.0 ± 0.3 ^c^	1.0 ± 0.4 ^c^
		1.8–4.7	0.8–3.6	1.5–5.3	0.9–5.0	0.5–1.8	0.4–2.5	0.5–1.7	0.6–1.8
*Synechocystis* PCC6803	14	3.7 ± 1.2 ^ab^	5.2 ± 3.5 ^b^	3.3 ± 3.1 ^a^	1.3 ± 0.6 ^ac^	1.0 ± 0.4 ^c^	1.0 ± 0.4 ^c^	1.0 ± 0.8 ^c^	1.0 ± 0.6 ^c^
		2.1–6.8	2.7–13.6	1.2–10.5	0.6–2.5	0.5–1.7	0.4–1.9	0.4–2.9	0.5–2.9
*P. agardhii* No371/1	24	1.4 ± 1.3 ^b^	1.7 ± 0.6 ^a^	1.2 ± 0.6 ^b^	1.6 ± 0.3 ^a^	1.0 ± 1.1 ^b^	1.0 ± 0.2 ^b^	1.0 ± 0.2 ^b^	1.0 ± 0.4 ^b^
		0.2–4.7	0.9–3.4	0.3–2.7	1.2–2.4	0.2–3.6	0.6–1.4	0.6–1.3	0.4–2.0
*P. agardhii* CYA126/8 WT	38	0.9 ± 0.2 ^b^	0.9 ± 0.2 ^b^	0.9 ± 0.2 ^b^	1.3 ± 0.2 ^a^	1.0 ± 0.2 ^b^	1.0 ± 0.2 ^b^	1.0 ± 0.3 ^b^	1.0 ± 0.3 ^b^
		0.5–1.2	0.4–1.2	0.6–1.1	0.7–1.7	0.7–1.4	0.6–1.4	0.6–1.7	0.2–1.8
*P. agardhii* CYA126/8 ΔapnC	40	n/d	1.3 ± 0.3 ^ab^	1.1 ± 0.2 ^a^	1.0 ± 0.2 ^b^	n/d	1.0 ± 0.2 ^a^	1.0 ± 0.3 ^a^	1.0 ± 0.2 ^a^
			0.6–2.0	0.6–1.5	0.6–1.3		0.7–1.5	0.3–1.5	0.7–1.3
*P. agardhii* CYA126/8 ΔociA	39	2.4 ± 1.1 ^a^	2.1 ± 0.3 ^a^	2.6 ± 0.5 ^a^	1.9 ± 0.5 ^a^	1.0 ± 0.2 ^b^	1.0 ± 0.4 ^b^	1.0 ± 0.3 ^b^	1.0 ± 0.3 ^b^
		1.1–5.5	1.6–2.9	2.0–4.2	1.0–2.8	0.6–1.1	0.5–2.2	0.2–2.0	0.6–1.7
*P. agardhii* CYA126/8 ΔmvdC	40	2.6 ± 1.5 ^ab^	2.7 ± 0.7 ^a^	2.2 ± 0.5 ^a^	1.3 ± 0.6 ^b^	1.0 ± 0.3 ^b^	1.0 ± 0.3 ^b^	1.0 ± 0.2 ^b^	1.0 ± 0.2 ^b^
		0.5–5.4	1.5–4.5	1.5–3.2	0.7–3.5	0.6–1.5	0.7–1.8	0.7–1.3	0.4–1.4
*P. agardhii* CYA126/8 ΔmcyD	6	0.9 ± 0.1 ^b^	2.6 ± 0.4 ^a^	3.5 ± 0.8 ^a^	2.1 ± 0.5 ^a^	1.0 ± 0.4 ^b^	1.0 ± 0.4 ^b^	1.0 ± 0.3 ^b^	1.0 ± 0.2 ^b^
		0.8–1.1	1.5–3.4	1.6–4.5	1.3–3.6	0.7–1.9	0.6–1.8	0.7–1.7	0.6–1.5

^1^ Kruskal–Wallis ANOVA on Ranks. Superscripts indicate homogeneous subgroups not significantly different at *p* < 0.05 using post-hoc pairwise comparison (Tukey’s test); n/d: no data.

## Data Availability

Data supporting this study, as well as the code used for generating the figures can be found in https://github.com/RubenMoronAsensio/Chem_modif_peptides (accessed on 22 July 2021).

## Supplementary Materials

The following are available online at https://www.mdpi.com/2076-2607/9/8/1578/s1, Table S1: Growth rate and collected dry biomass of the strains used in this study. Figure S1: Workflow diagram describing the processes applied for this study. Figure S2: Confocal images of modified MCs (*M. aeruginosa*) or APs (*P. agardhii*) obtained from cells supplemented with Phe-Az. Figure S3: Confocal images of modified MCs (*M. aeruginosa*) or APs (*P. agardhii*) obtained from cells supplemented with Prop-Lys. Figure S4: Confocal images of modified MCs (*M. aeruginosa*) or APs (*P. agardhii*) obtained from cells in absence or presence supplemented AA Phe-Az, Prop-Lys and Prop-Tyr. Figure S5: *M. aeruginosa* LC-MS BPC and EIC chromatograms. Figure S6: *Synechocystis* PCC6803 LC-MS BPC and EIC chromatograms. Figure S7: *P. agardhii* No371/1 LC-MS BPC and EIC chromatograms. Figure S8: *P. agardhii* CYA126/8 WT LC-MS BPC and EIC chromatograms. Figure S9: *P. agardhii* CYA126/8 ΔapnC LC-MS BPC and EIC chromatograms. Figure S10: *P. agardhii* CYA126/8 ΔociA LC-MS BPC and EIC chromatograms. Figure S11: *P. agardhii* CYA126/8 ΔmvdC LC-MS BPC and EIC chromatograms. Figure S12: *P. agardhii* CYA126/8 ΔmcyD LC-MS BPC and EIC chromatograms. Figure S13: Confocal images of non-modified MCs (*M. aeruginosa*) or APs (*P. agardhii*) and lipids. Figure S14: Confocal images of lipids from cells supplemented with Phe-Az. Figure S15: Confocal images of simultaneous labeling of Prop-Lys modified MCs (*M. aeruginosa*) or APs (*P. agardhii*) and lipids used for colocalization measurements. Figure S15: Confocal images of simultaneous labeling of Prop-Lys modified MCs (*M. aeruginosa*) or APs (*P. agardhii*) and lipids used for colocalization measurements.
