# Correction: Morón-Asensio et al. Differential Labeling of Chemically Modified Peptides and Lipids among Cyanobacteria *Planktothrix* and *Microcystis*. *Microorganisms* 2021, *9*, 1578

**DOI:** 10.3390/microorganisms10040695

**Published:** 2022-03-24

**Authors:** Rubén Morón-Asensio, David Schuler, Anneliese Wiedlroither, Martin Offterdinger, Rainer Kurmayer

**Affiliations:** 1 Research Department for Limnology, University of Innsbruck, Mondseestrasse 9, 5310 Mondsee, Austria; David.schuler@uibk.ac.at (D.S.); anneliese.wiedlroither@uibk.ac.at (A.W.); 2 Core Facility Biooptics (CCB), Medical University Innsbruck, Innrain 80-82, 6020 Innsbruck, Austria; martin.offterdinger@i-med.ac.at

The authors wish to make the following corrections to this paper [1]:

We repeated the peptide analysis as well as labeling for the *P. agardhii* CYA126/8 mutant strain with experimentally inactivated AP synthesis (ΔapnC), since, as reported, the ΔapnC mutant strain did not only stop producing anabaenopeptins (APs) but, unexpectedly, also did not contain microcystins (MCs) (Figure S9). Thus, in comparison with the WT strain CYA126/8, it was impossible to find out whether, besides Aps, unnatural amino acids Prop-Tyr, Prop-Lys, or Phe-Az might be incorporated into the MC molecule during its synthesis and contribute to the observed labeling signal (Figure 3, Figure 4 and Table 2, Table 3). 

To elucidate the possible incorporation of unnatural amino acids into the MCs strain, *P. agardhii* CYA126/8 ΔapnC was regrown from one single filament from the original mutant culture [2] and reanalyzed under identical conditions. Peptide analysis via LC-MS revealed that both AP 905 and AP 915 were lacking but as expected, the other peptides, i.e., aeruginosin 126A, 126B, Microviridin K, demethylated MC-RR, demethylated MC-LR, cyanopeptolin 880, and sulfated cyanopeptolin 960 were still produced (corrected Figure S9 as follows).

In addition, during the reanalysis of the ΔapnC strain peptide extract, no clear evidence for incorporation of Prop-Tyr, Prop-Lys, or Phe-Az into the MC molecules was observed (corrected Figure S9 as follows). As for *M. aeruginosa* (Table 1), a modified D-Asp-MC-Tyr-alkyne [M + H] 1069.5 has been predicted from the original MC molecular weight, subtracting the mass of the original AA (Htyr = 195.2), and adding the mass of the non-natural AA (Prop-Tyr = 219.2) added, but could not be unequivocally identified (Figure S9A).

Nevertheless, some increased intensity for Prop-Tyr was observed using ALEXA488 (1.3 ± 0.4 vs. 0.7 ± 0.2, *p* < 0.001) and ALEXA405 (1.4 ± 0.2 vs. 0.6 ± 0.1, *p* < 0.05) while no increase was detected for the Prop-Lys fed cultures labeled with ALEXA405. However, since this increase in intensity was rather small, the ΔapnC mutant did not show increased ratios of ALEXA488 to autofluorescence (AF) and ALEXA405 to AF under Prop-Tyr feeding conditions (corrected Figure 3 and Figure 4). 

Thus, we conclude that the increase in ALEXA488 or ALEXA405 intensity for the *P. agardhii* ΔapnC mutant strain was not derived from D-Asp-MC-Tyr-alkyne [M + H] 1069.5 to a major extent. Since neither the ALEXA488 nor the ALEXA405 fluorescence to AF ratios were affected, we conclude that the signal increase from Tyr-alkyne was relatively minor.

**Figure S9:**
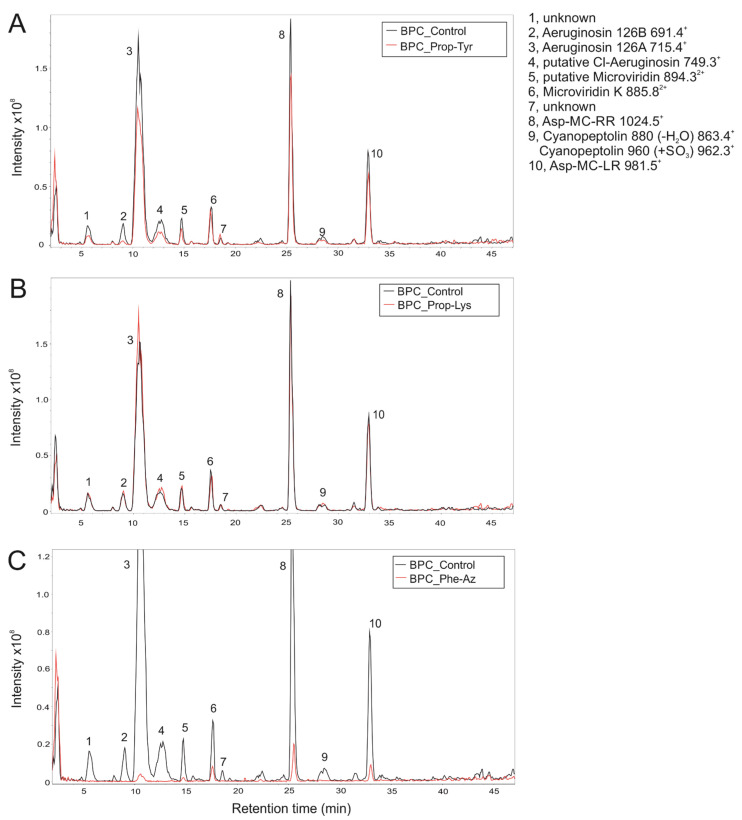
LC-MS Base Peak Chromatograms (BPC) and Extracted Ion Chromatograms (EIC) in positive ionization mode for *P. agardhii* strain CYA126/8 ΔapnC mutant insertionally inactivated in AP synthesis [2]. *P. agardhii* was grown in the presence of (**A**) Prop-Tyr, (**B**) Prop-Lys, and (**C**) Phe-Az. Controls were from cells grown under identical conditions but without substrate. As expected, this mutant strain did not contain any AP molecule but the other peptide groups, i.e., aeruginosin 126A, 126B, Microviridin K, demethylated MC-RR, demethylated MC-LR, cyanopeptolin 880, and sulfated cyanopeptolin 960.

A correction of the original version follows:

In Section 3.2.1 Peptide Labeling Intensity, the fourth and fifth sentence of the fourth paragraph should read as follows: “In comparison with CYA126/8 WT, labeling intensity was found to be less reduced in the AP synthesis mutant ΔapnC, i.e., a slightly increased intensity for Prop-Tyr was observed using ALEXA488 (1.3 ± 0.4 vs. 0.7 ± 0.2, *p* < 0.001) and ALEXA405 (1.4 ± 0.2 vs. 0.6 ± 0.1, *p* < 0.05) while no increase was detected for the Prop-Lys fed cultures labeled with ALEXA405.”

In Section 3.2.2 Peptide Intensity/Autofluorescence Ratio, the last sentence of the second paragraph should read as follows: “The ΔapnC mutant did not show increased ratios of peptide intensity vs. AF between the treatments.”

In Section 3.2.2 Peptide Intensity/Autofluorescence Ratio, the fourth sentence of the fourth paragraph should read as follows: “Using ALEXA405 labeling, the ΔapnC mutant did not reveal a change in signal ratio, i.e., the median ratio varied from 1.7 (Prop-Lys), 1.9 (Prop-Tyr), and 1.7 (Control).”

In Section 4 Discussion, the third paragraph should read as follows: “Currently the observed, rather moderate, Prop-Tyr labeling for the *P. agardhii* ΔapnC mutant strain does not support our hypothesis on Prop-Tyr incorporation during MC biosynthesis, i.e., identification of its derivation from D-Asp-MC-Tyr-alkyne [M + H] 1069.5 could not be unequivocally performed. However, since neither the ALEXA488 nor the ALEXA405 fluorescence to AF ratios were affected, we conclude that the signal increase from Tyr-alkyne in the ΔapnC mutant strain was generally minor.”

Additionally, the following tables and figures have been updated to represent the results obtained for the corrected *P. agardhii* ΔapnC strain analysis results.

**Figure 3:**
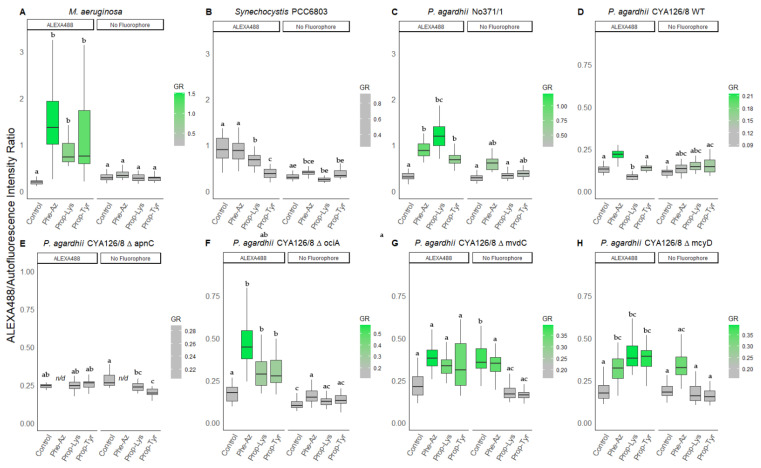
The ratio of green fluorescence intensity (ALEXA488) to red intensity (AF) for individual cells or filaments from (**A**) *M. aeruginosa*, (**B**) *Synechocystis* PCC6803, and (**C**) *P. agardhii* strain No371/1, (**D**) CYA126/8 WT, and (**E**–**H**) CYA126/8 gene knock out mutants: (**E**) ΔapnC (inactivated AP synthesis), (**F**) ΔociA (inactivated cyanopeptolin synthesis), (**G**) ΔmvdC (inactivated microviridin synthesis), and (**H**) ΔmcyD (inactivated MC synthesis) using non-natural amino acids (Phe-Az, Prop-Lys, and Prop-Tyr). Controls were grown without amino acid addition but used for the chemical reaction under identical conditions. No Fluorophore indicates filaments or cells grown with amino acid addition but no subsequent labeling by click-chemical reaction. The gradient in coloring was defined for each strain separately using the average intensity from the control cultures. Superscripts indicate statistically significant different subgroups after overall difference was found (*p* < 0.05).

**Figure 4:**
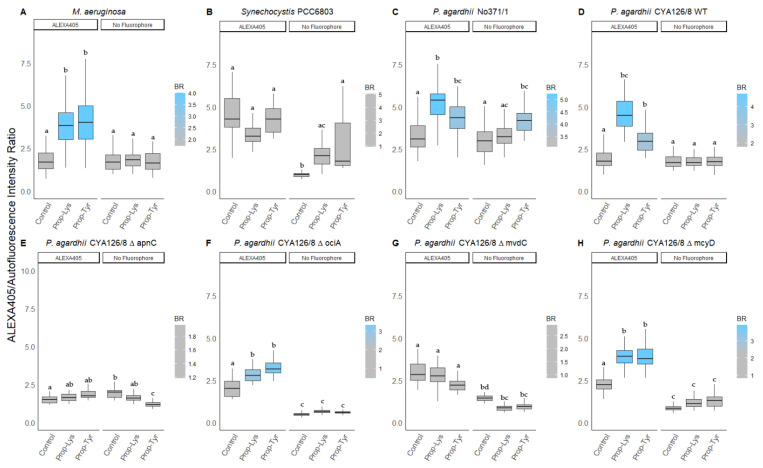
The ratio of blue fluorescence intensity (ALEXA405) to red intensity (AF) for individual cells or filaments from (**A**) *M. aeruginosa*, (**B**) *Synechocystis* PCC6803, and (**C**) *P. agardhii* strain No371/1, (**D**) CYA126/8 WT, and (**E**–**H**) CYA126/8 gene knock out mutants: (**E**) ΔapnC (inactivated AP synthesis), (**F**) ΔociA (inactivated cyanopeptolin synthesis), (**G**) ΔmvdC (inactivated microviridin synthesis), and (**H**) ΔmcyD (inactivated MC synthesis) using non-natural amino acids (Phe-Az, Prop-Lys, and Prop-Tyr). Controls were grown without amino acid addition but used for the chemical reaction under identical conditions. No Fluorophore indicates filaments or cells grown with amino acid addition but no subsequent labeling by click-chemical reaction. The gradient in coloring was defined for each strain separately using the average intensity from the control cultures. Superscripts indicate statistically significant different subgroups after overall difference was found (*p* < 0.05).

**Table 2:** Average (±SD) min–max green fluorescence intensity obtained for individual treatments using non-natural amino acid feeding (Phe-Az, Prop-Lys, and Prop-Tyr) and subsequent labeling by ALEXA488 using copper-catalyzed azid-alkyne cycloaddition (CuAAC). The intensity was divided by the average intensity of control filaments or cells, i.e., cells which were grown without amino acid addition but used for the chemical reaction under identical conditions. No Fluorophore indicates filaments or cells grown with amino acid addition but no subsequent labeling by the click-chemical reaction. n: number of individual filaments (*Planktothrix*) or cells (*Microcystis*, *Synechocystis*).

		ALEXA488 ^1^			No Fluorophore ^1^		
	n	Phe-Az	Prop-Lys	Prop-Tyr	Phe-Az	Prop-Lys	Prop-Tyr
*M. aeruginosa* Hofbauer	43	4.6 ± 2.2 ^a^	4.4 ± 3.7 ^a^	5.3 ± 2.6 ^a^	1.2 ± 0.3 ^b^	1.0 ± 0.3 ^b^	1.0 ± 0.3 ^b^
		1.1–12.5	1.6–19.4	1.1–13.0	0.6–1.7	0.5–1.6	0.5–1.6
*Synechocystis* PCC6803	20	1.0 ± 0.8 ^ab^	0.5 ± 0.6 ^a^	0.3 ± 0.1 ^a^	1.5 ± 0.6 ^b^	2.1 ± 0.8 ^bc^	2.4 ± 1.8 ^bc^
		0.3–3.2	0.2–2.7	0.1–0.4	0.8–2.6	0.8–4.0	0.9–6.9
*P. agardhii* No371/1	16	1.3 ± 0.6 ^a^	4.0 ± 1.2 ^b^	1.9 ± 0.4 ^c^	0.6 ± 0.7 ^a^	0.8 ± 0.2 ^a^	0.9 ± 0.2 ^a^
		0.5–3.3	2.1–7.5	1.3–2.7	0.1–2.2	0.5–1.2	0.6–1.2
*P. agardhii* CYA126/8 WT	38	2.0 ± 0.5 ^b^	1.3 ± 0.3 ^ac^	1.6 ± 0.2 ^ad^	1.2 ± 0.2 ^a^	1.2 ± 0.3 ^a^	1.4 ± 0.5 ^a^
		0.5–2.5	0.8–1.9	0.8–1.9	0.8–1.7	0.8–1.7	0.8–2.3
*P. agardhii* CYA126/8 ΔapnC	20	n/d	1.0 ± 0.1^a^	1.3 ± 0.4 ^b^	n/d	0.9 ± 0.2 ^ac^	0.7 ± 0.2 ^c^
			0.8–1.3	1.0–2.6		0.7–1.3	0.5–1.1
*P. agardhii* CYA126/8 ΔociA	38	1.8 ± 0.5 ^a^	2.5 ± 0.7 ^a^	2.4 ± 0.6 ^a^	1.0 ± 0.2 ^b^	1.1 ± 0.5 ^b^	1.0 ± 0.3 ^b^
		1.0–3.1	1.5–3.6	1.4–3.6	0.6–1.3	0.6–2.5	0.2–2.0
*P. agardhii* CYA126/8 ΔmvdC	40	0.9 ± 0.3 ^a^	1.7 ± 0.3 ^ab^	1.5 ± 0.7 ^a^	0.6 ± 0.2 ^c^	0.6 ± 0.2 ^c^	0.7 ± 0.1 ^c^
		0.6–1.9	1.2–2.4	0.6–2.5	0.3–0.9	0.4–1.1	0.5–0.8
*P. agardhii* CYA126/8 ΔmcyD	39	0.9 ± 0.3 ^b^	1.7 ± 0.3 ^a^	1.6 ± 0.3 ^a^	1.0 ± 0.3 ^b^	0.9 ± 0.3 ^b^	0.9 ± 0.2 ^b^
		0.5–1.9	1.3–2.3	1.0–2.2	0.6–1.7	0.5–1.5	0.6–1.5

^1^ For each strain, treatments were compared using Kruskal–Wallis ANOVA on Ranks. We found statistically significant differences between the treatments (*p* < 0.001) in all of them. Superscripts indicate homogeneous subgroups not significantly different at *p* < 0.05 using post-hoc pairwise comparison (Tukey’s test); n/d: no data.

**Table 3:** Average (±SD) min–max blue fluorescence intensity obtained for individual treatments using non-natural amino acid feeding (Prop-Lys and Prop-Tyr) and subsequent labeling by ALEXA405 using copper-catalyzed azide-alkyne cycloaddition (CuAAC). The intensity was divided by the average intensity of control filaments or cells, i.e., cells were grown without amino acid addition but used for the chemical reaction under identical conditions. No Fluorophore indicates filaments or cells grown with amino acid addition but no subsequent labeling by the click-chemical reaction. n: number of individual filaments (*Planktothrix*) or cells (*Microcystis, Synechocystis*).

		ALEXA405 ^1^		No Fluorophore ^1^	
	(n)	Prop-Lys	Prop-Tyr	Prop-Lys	Prop-Tyr
*M. aeruginosa* Hofbauer	50	1.9 ± 0.6 ^a^	2.4 ± 0.9 ^a^	1.1 ± 0.4 ^bc^	1.0 ± 0.4 ^b^
		0.8–3.5	0.5–3.7	0.4–2.1	0.3–1.9
*Synechocystis* PCC6803	14	0.9 ± 0.6 ^a^	0.9 ± 0.3 ^a^	5.5 ± 2.4 ^b^	5.6 ± 5.3 ^b^
		0.3–2.1	0.4–1.5	2.1–9.7	1.6–19.8
*P. agardhii* No371/1	39	1.2 ± 0.2 ^a^	0.9 ± 0.3 ^a^	0.8 ± 0.2 ^ab^	1.0 ± 0.2 ^a^
		0.7–1.7	0.4–1.5	0.5–1.1	0.6–1.4
*P. agardhii* CYA126/8 WT	38	2.3 ± 0.5 ^c^	1.6 ± 0.3 ^a^	0.9 ± 0.2 ^b^	1.0 ± 0.2 ^b^
		1.3–3.6	0.9–2.2	0.6–1.5	0.7–1.5
*P. agardhii* CYA126/8 ΔapnC	20	1.0 ± 0.2 ^a^	1.4 ± 0.2 ^b^	0.9 ± 0.1 ^a^	0.6 ± 0.1 ^d^
		0.7–1.3	1.0–1.9	0.8–1.1	0.5–0.8
*P. agardhii* CYA126/8 ΔociA	39	1.4 ± 0.3 ^ac^	1.6 ± 0.3 ^a^	0.7 ± 0.2 ^bc^	1.0 ± 0.3 ^b^
		0.9–2.0	1.2–2.4	0.7–2.2	0.4–1.7
*P. agardhii* CYA126/8 ΔmvdC	40	1.1 ± 0.3 ^a^	1.0 ± 0.2 ^a^	0.8 ± 0.2 ^c^	1.0 ± 0.2 ^a^
		0.6–1.7	0.6–1.4	0.5–1.3	0.8–1.3
*P. agardhii* CYA126/8 ΔmcyD	38	1.5 ± 0.2 ^a^	1.3 ± 0.3 ^a^	1.2 ± 0.3 ^abd^	1.5 ± 0.3 ^ab^
		1.0–1.9	0.8–1.8	0.9–2.0	0.8–2.2

^1^ For each strain, treatments were compared using Kruskal–Wallis ANOVA on Ranks. We found statistically significant differences between the treatments (*p* < 0.001) in all of them. Superscripts indicate homogeneous subgroups not significantly different at *p* < 0.05 using post-hoc pairwise comparison (Tukey’s test).

Change in Supplementary Materials:

The Supplementary Materials were changed accordingly and were included as a separate document: https://www.mdpi.com/article/10.3390/microorganisms9081578/s1.

The authors would like to apologize for any inconvenience caused to the readers by these changes.

## References

[1] Morón-Asensio R., Schuler D., Wiedlroither A., Offterdinger M., Kurmayer R. (2021). Differential Labeling of Chemically Modified Peptides and Lipids among Cyanobacteria Planktothrix and Microcystis. Microorganisms.

[2] Christiansen G., Philmus B., Hemscheidt T., Kurmayer R. (2011). Genetic variation of adenylation domains of the anabaenopeptin synthesis operon and evolution of substrate promiscuity. J. Bacteriol..

