# Supplementary material for: Differential Labeling of Chemically Modified Peptides and Lipids among Cyanobacteria Planktothrix and Microcystis
Source: Microorganisms. 2021 Jul 24;9(8):1578. doi: 10.3390/microorganisms9081578 (PMC8398151; doi:10.3390/microorganisms9081578)
Supplement: Supplementary file 1 [file microorganisms-09-01578-s001.zip › microorganisms-1263867-supplementary/microorganisms-1263867-supplementary.pdf]

Differential Labeling of Chemically Modified Peptides and Lipids among Cyanobacteria *Planktothrix* and *Microcystis*.

Rubén Morón-Asensio[1], David Schuler[1], Anneliese Wiedlroither[1], Martin Offterdinger[2] and Rainer Kurmayer[1*]

[1] Research Department for Limnology, University of Innsbruck, Mondseestrasse 9, 5310, Mondsee, Austria; rainer.kurmayer@uibk.ac.at
[2] Core Facilitiy Biooptics (CCB), Medical University Innsbruck, Innrain 80-82, 6020, Innsbruck, Austria
* Correspondence: rainer.kurmayer@uibk.ac.at

Supplementary material.

Table S1. Growth rate of the cultures under semicontinuous growth conditions following the turbidostat principle in the presence of non-natural amino acids and in the absence (control). Dry weight (DW) used for peptide analysis by LC-MS is indicated.

	**Phe-Az**		**Prop-Lys**		**Prop-Tyr**		**Control**		**Pre-culture ***
	Growth rate d⁻¹	*Dry weight (mg)*	*Growth rate d⁻¹*	*Dry weight (mg)*	*Growth rate d⁻¹*	*Dry weight (mg)*	*Growth rate d⁻¹*	*Dry weight (mg)*	*Growth rate d⁻¹*
M. aeruginosa Hofbauer	0.32[a]	0.88	0.56[a]	1.54	0.55[a]	0.86**	0.57[a]	1.38	0.53[a] ± 0.17
Synechocystis PCC6803	0.25[b]	0.65	0.96[a]	1.63	0.96[a]	1.49	1.01[a]	1.85	0.87[a] ± 0.24
P. agardhii No371/1	0.22[a]	0.46	0.38[a]	1.78	0.38[a]	1.86	0.37[a]	1.86	0.47[a] ± 0.22
P. agardhii CYA126/8 WT	0.26[b]	0.99	0.44[a]	2.69	0.45[a]	2.58[a]	0.47[a]	2.98	0.46[a] ± 0.10
P. agardhii CYA126/8 *ΔapnC*	0.13[b]	0.93	0.36[a]	2.72	0.32[a]	2.90	0.32[a]	2.40	0.43[a] ± 0.11
P. agardhii CYA126/8 *ΔociA*	0.28[a]	1.38	0.35[a]	2.75	0.43[a]	2.45	0.46[a]	3.01	0.47[a] ± 0.22
P. agardhii CYA126/8 *ΔmvdC*	0.24[b]	1.36	0.27[a]	2.83	0.36[a]	2.86	0.37[a]	2.99	0.47[a] ± 0.13
P. agardhii CYA126/8 *ΔmcyD*	0.21[b]	1.12	0.42[a]	4.04	0.41[a]	3.72	0.40[a]	4.07	0.49[a] ± 0.12

[1] Z-test assuming normal distribution. Superscripts indicate homogeneous subgroups not significantly different at p<0.05. *Precultures were maintained under semicontinuous growth conditions [1]. **Loss of vacuum due to a faulty seal caused the approximately loss of 15ml the culture.

Figure S1. Workflow in peptide labeling of cyanobacteria used in this study. Precultures were growth following the turbidostat principle until they reached an optical density (OD_{600nm}) if 0.1. Once the precultures reached an OD_{600nm} of 0.1 cultures were separated into three new culture flasks per feeding treatment, i.e. either 0.05 mM Phe-Az, Prop-Lys, Prop-Tyr dissolved in 1mM NaOH or control cultures, which were supplied with 1 mM NaOH only. Flask 1 was used for daily growth rate measurements, taken at regular intervals. Flask 2 was filtered using GF/C filters, biomass obtained from the filters was dried in a vacuum centrifuge and peptide content was extracted using aqueous MeOH (50%, *v/v*). Extracted peptides were then analyzed via HPLC-MS. From flask 3 cells were harvested by centrifugation, fixed with 2% PFA and subsequently labeled: Without addition of any fluorophore but processed in the same way; labeled with ALEXA488 & ALEXA405, for lipids stained with BODIPY 505/515, followed by peptide labeling with ALEXA405. Labeled samples were transferred onto microscopy slides with ProLong Diamond Antifade mountant as a preserving agent. Microscopic images were taken using a confocal microscope Leica Microsystems SP8 and analyzed using Huygens Essential 20.04 and data was processed and visualized using RStudio 4.01

Figure S2. Confocal microscopy images from cells fed with Phe-Az modified MCs (*M. aeruginosa*) or APs (*P. agardhii* No371/1 and CYA126/8 and peptide knock out mutants). *Synechocystis* PCC6803 (lacking NRPS) was used as additional control. Cells were grown in the presence of Phe-Az and labeled with ALEXA488 (A488) and since ALEXA405 (A405) only was available as an azide but not as an alkyne it could not be applied for MC or AP labeling carrying the Phe-Az moiety, while AF indicates autofluorescence.

Figure S3. Confocal microscopy images from cells fedf with Prop-Lys modified MCs (*M. aeruginosa*) or APs (*P. agardhii* No371/1 and CYA126/8 and peptide knock out mutants). *Synechocystis* PCC6803 (lacking NRPS) was used as additional control. Cells were grown in the presence of Prop-Lys and labeled with ALEXA488 (A488) and ALEXA405 (A405) while AF indicates autofluorescence.

Figure S4. Confocal microscopy images from cells with modified MCs (*M. aeruginosa*) or APs (*P. agardhii* No371/1 and CYA126/8 and peptide knock out mutants) with non-natural amino acids. *Synechocystis* PCC6803 (lacking NRPS) was used as additional control. Cells were grown in the presence or absence (Control) of different non-natural amino acids (Phe-Az, Prop-Lys or Prop-Tyr) and labeled with ALEXA488 (A488) and ALEXA405 (A405). Since ALEXA405 only was available as an azide but not as an alkyne it could not be applied for MC or AP labeling carrying the Phe-Az moiety, while AF indicates autofluorescence; n/a; not applicable.

Figure S5. LC-MS Base Peak Chromatograms (BPC) and Extracted Ion Chromatograms (EIC) in positive ionization mode for *M. aeruginosa* strain Hofbauer showing the synthesis of new MC structural variants carrying the alkyne moiety or azide moiety to be used for click chemistry reactions, such as copper-catalyzed azide-alkyne cycloaddition. *M. aeruginosa* was grown in the presence of (**A**) Prop-Tyr, (**B**) Prop-Lys, (**C**) Phe-Az. Controls from cells grown under identical conditions but without substrate. EIC 220.0, EIC 229.0 and EIC 207.0 indicate the elution of Prop-Tyr (6 min), Prop-Lys (3.4 min) and Phe-Az (5.9 min) respectively.

Figure S6. LC-MS Base Peak Chromatograms (BPC) and Extracted Ion Chromatograms (EIC) in positive ionization mode for *Synechocystis* strain PCC6803 grown in the presence of (**A**) Prop-Tyr, (**B**) Prop-Lys, (**C**) Phe-Az. Controls from cells grown under identical conditions but without substrate. EIC 220.0, EIC 229.0 and EIC 207.0 indicate the elution of Prop-Tyr (6 min), Prop-Lys (3.4 min) and Phe-Az (6 min), respectively.

Figure S7. LC-MS Base Peak Chromatograms (BPC) and Extracted Ion Chromatograms (EIC) in positive ionization mode for *P. agardhii* strain No371/1 showing the synthesis of new AP structural variants carrying the alkyne moiety or azide moiety to be used for click chemistry reactions, such as copper-catalyzed azide-alkyne cycloaddition. *P. agardhii* was grown in the presence of (**A**) Prop-Tyr, (**B**) Prop-Lys, (**C**) Phe-Az. Controls from cells grown under identical conditions but without substrate. EIC 220.0, EIC 229.0 and EIC 207.0 indicate the elution of Prop-Tyr (6 min), Prop-Lys (3.3 min) and Phe-Az (6.1 min) respectively.

Figure S8. LC-MS Base Peak Chromatograms (BPC) and Extracted Ion Chromatograms (EIC) in positive ionization mode for *P. agardhii* strain CYA126/8 showing the synthesis of new AP structural variants carrying the alkyne moiety or azide moiety to be used for click chemistry reactions, such as copper-catalyzed azide-alkyne cycloaddition. *P. agardhii* was grown in the presence of (**A**) Prop-Tyr, (**B**) Prop-Lys, (**C**) Phe-Az. Controls from cells grown under identical conditions but without substrate. EIC 220.0, 229.0 and 207.0 indicate the elution of Prop-Tyr (6 min), Prop-Lys (3.3 min) and Phe-Az (6 min) respectively.

Figure S9. LC-MS Base Peak Chromatograms (BPC) and Extracted Ion Chromatograms (EIC) in positive ionization mode for *P. agardhii* strain CYA126/8 *ΔapnC* mutant insertionally inactivated in AP synthesis [2]. *P. agardhii* was grown in the presence of (**A**) Prop-Tyr, (**B**) Prop-Lys, and (**C**) Phe-Az. Controls were from cells grown under identical conditions but without substrate. As expected, this mutant strain did not contain any AP molecule but the other peptide groups, i.e., aeruginosin 126A, 126B, Microviridin K, demethylated MC-RR, demethylated MC-LR, cyanopeptolin 880, and sulfated cyanopeptolin 960.

Figure S10. LC-MS Base Peak Chromatograms (BPC) and Extracted Ion Chromatograms (EIC) in positive ionization mode for *P. agardhii* strain CYA126/8 ΔociA mutant insertionally inactivated in cyanopeptolin synthesis (unpublished data). *P. agardhii* was grown in the presence of (**A**) Prop-Tyr, (**B**) Prop-Lys, (**C**) Phe-Az. Controls from cells grown under identical conditions but without substrate. As expected this mutant strain did not contain any cyanopeptolin molecule. EIC 220.0, and EIC 207.0 indicate the elution of Prop-Tyr (5.9 min), and Phe-Az (5.9 min) respectively. Relatively little elution of EIC 229.0 indicative of Prop-Lys (3.3 min) was found.

Figure S11. LC-MS Base Peak Chromatograms (BPC) and Extracted Ion Chromatograms (EIC) in positive ionization mode for *P. agardhii* strain CYA126/8 ΔmvdC mutant insertionally inactivated in microviridin synthesis [3]. *P. agardhii* was grown in the presence of (**A**) Prop-Tyr, (**B**) Prop-Lys, (**C**) Phe-Az. Controls from cells grown under identical conditions but without substrate. As expected this mutant strain did not contain any microviridin molecule. EIC 220.0, EIC 229.0 and EIC 207.0 indicate the elution of Prop-Tyr (5.9 min), Prop-Lys (3.4 min) and Phe-Az (5.9 min) respectively.

Figure S12. LC-MS Base Peak Chromatograms (BPC) and Extracted Ion Chromatograms (EIC) in positive ionization mode for *P. agardhii* strain CYA126/8 ΔmcyD mutant insertionally inactivated in MC synthesis [4]. *P. agardhii* was grown in the presence of (**A**) Prop-Tyr, (**B**) Prop-Lys, (**C**) Phe-Az. Controls from cells grown under identical conditions but without substrate. As expected this mutant strain did not contain any MC molecule. EIC 220.0, EIC 229.0 and EIC 207.0 indicate the elution of Prop-Tyr (5.9 min), Prop-Lys (3.3 min) and Phe-Az (6.0 min) respectively.

Figure S13. Confocal microscopy images from control cells with non modified MC (*M. aeruginosa*) or AP (*P. agardhii* No371/1 and CYA126/8 and peptide knock out mutants) used for colocalization measurements as shown in Figure 6. *Synechocystis* PCC6803 (lacking NRPS) was used as additional control. Lipids were labeled using BODIPY 505/515 (BODIPY), subsequently modified peptides were labeled using ALEXA405 (A405). Controls were produced from cells without non natural amino acid feeding but processed under identical conditions. AF indicates autofluorescence.

Figure S14. Confocal microscopy images from cells fed with Phe-Az modified MC (*M. aeruginosa*) or AP (*P. agardhii* No371/1 and CYA126/8 and peptide knock out mutants) used for colocalization measurements as shown in Figure 6. *Synechocystis* PCC6803 (lacking NRPS) was used as additional control. Lipids were labeled using BODIPY 505/515 (BODIPY), while modified peptides could not be labeled using ALEXA405 (A405). AF indicates autofluorescence.

Figure S15. Confocal microscopy images from cells fed with Prop-Lys modified MC (*M. aeruginosa*) or AP (*P. agardhii* No371/1 and CYA126/8 and peptide knock out mutants) used for colocalization measurements as shown in Figure 6. *Synechocystis* PCC6803 (lacking NRPS) was used as additional control. Lipids were labeled using BODIPY 505/515 (BODIPY), subsequently modified peptides were labeled using ALEXA405 (A405) while AF indicates autofluorescence.

Figure S16. Confocal microscopy images from cells fed with Prop-Tyr modified MC (*M. aeruginosa*) or AP (*P. agardhii* No371/1 and CYA126/8 and peptide knock out mutants) used for colocalization measurements as shown in Figure 6. *Synechocystis* PCC6803 (lacking NRPS) was used as additional control. Lipids were labeled using BODIPY 505/515 (BODIPY), subsequently modified peptides were labeled using ALEXA405 (A405) while AF indicates autofluorescence.

References

1. Kohl, J.-G.; Nicklisch, A. *Ökophysiologie der Algen*: *Wachstum u. Ressourcennutzung;* Akad.-Verl.: Berlin, **1988**, ISBN 9783055003158.

2. Christiansen, G.; Philmus, B.; Hemscheidt, T.; Kurmayer, R. Genetic variation of adenylation domains of the anabaenopeptin synthesis operon and evolution of substrate promiscuity. *J. Bacteriol.* **2011**, *193*, 3822–3831, doi:10.1128/JB.00360-11.

3. Philmus, B.; Christiansen, G.; Yoshida, W.Y.; Hemscheidt, T.K. Post-translational modification in microviridin biosynthesis. *Chembiochem* **2008**, *9*, 3066–3073, doi:10.1002/cbic.200800560.

4. Kurmayer, R.; Christiansen, G. The Genetic Basis of Toxin Production in Cyanobacteria. *Freshwater Reviews* **2009**, *2*, 31–50, doi:10.1608/FRJ-2.1.2.
