# Supplementary figures and images for: Differential Labeling of Chemically Modified Peptides and Lipids among Cyanobacteria Planktothrix and Microcystis
Source: Microorganisms. 2021 Jul 24;9(8):1578. doi: 10.3390/microorganisms9081578 (PMC8398151; doi:10.3390/microorganisms9081578)

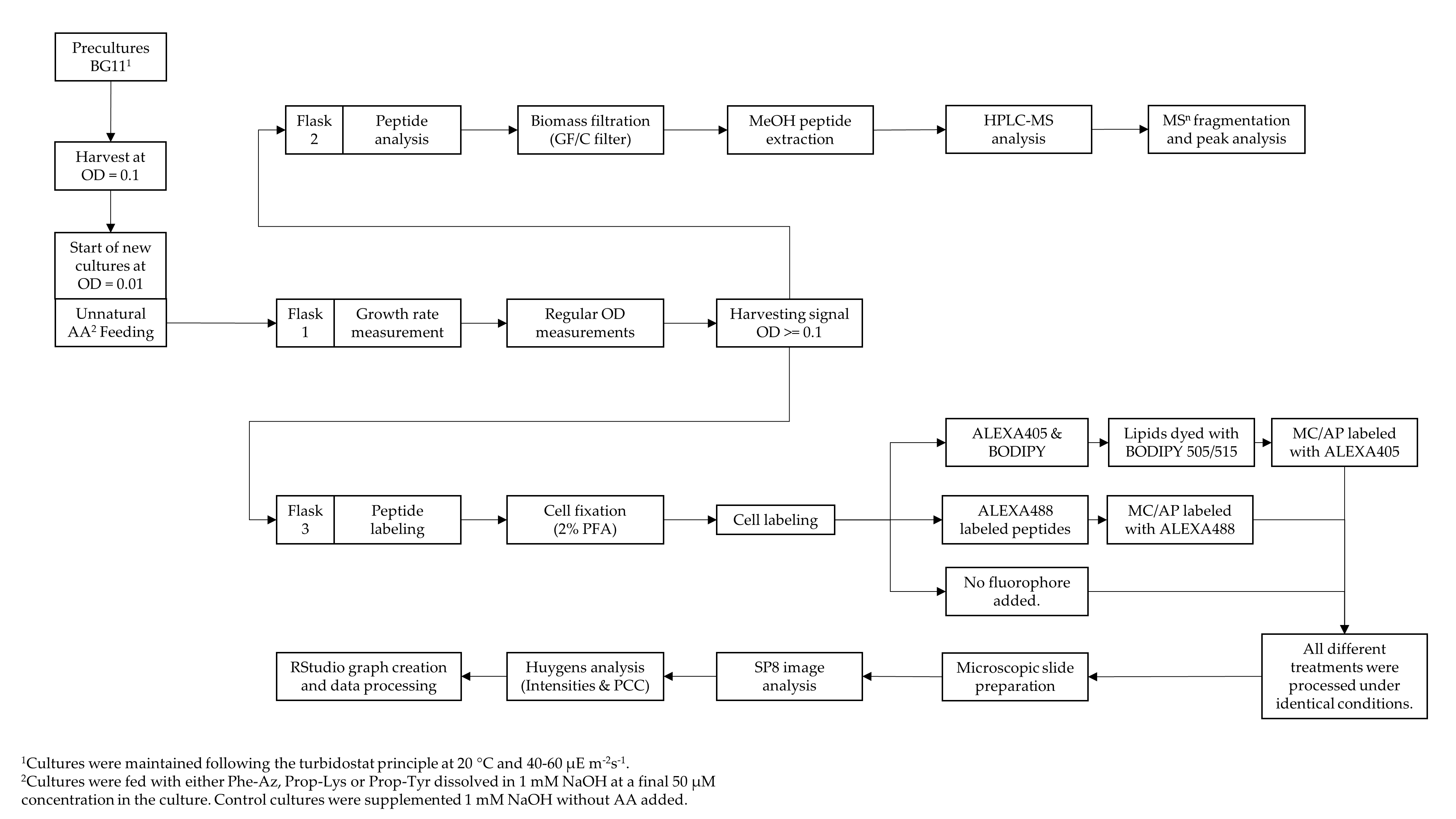

Supplement: Supplementary file 1 [file microorganisms-09-01578-s001.zip › microorganisms-1263867-supplementary/Figure S1.tif]

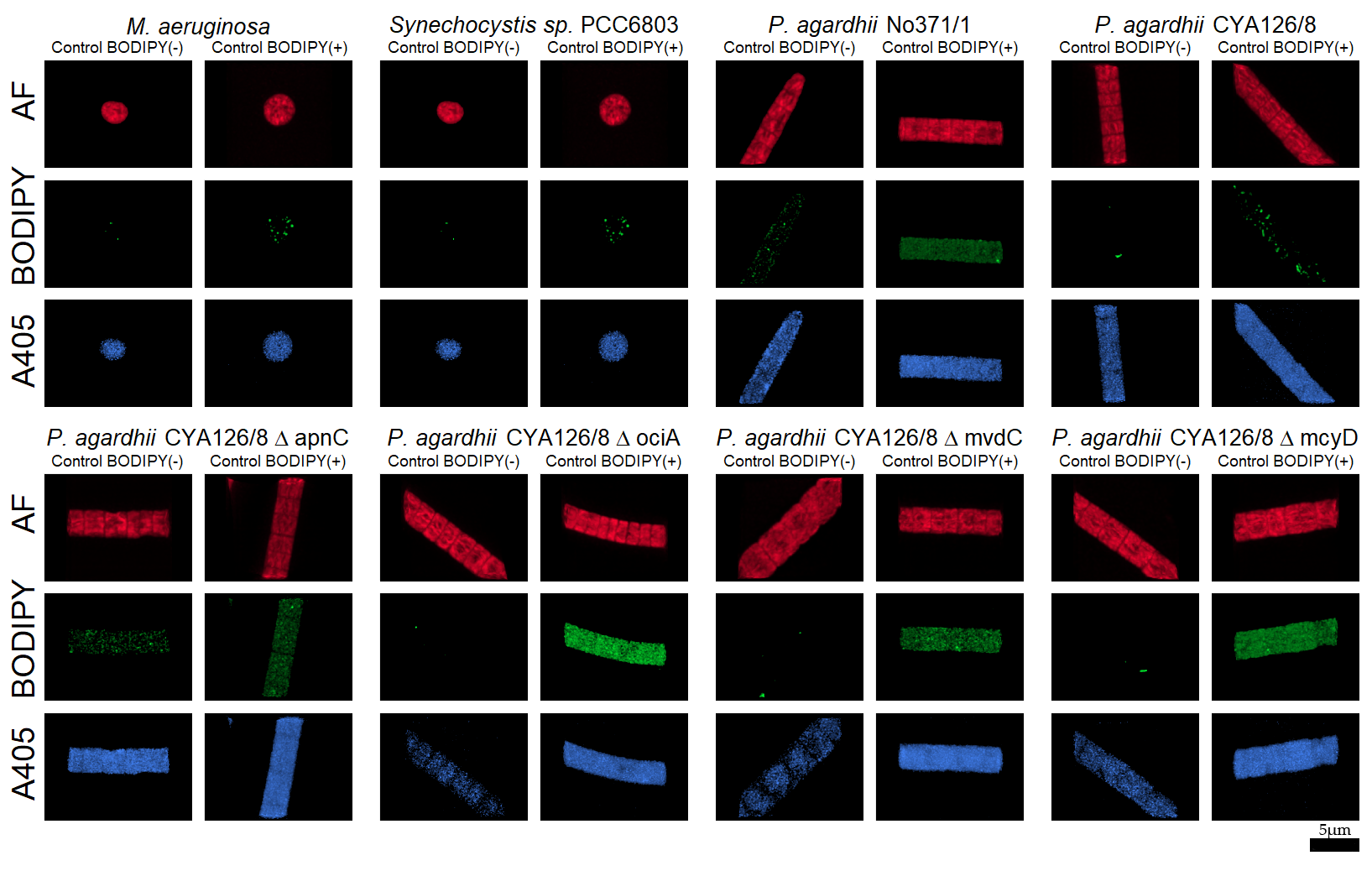

Supplement: Supplementary file 1 [file microorganisms-09-01578-s001.zip › microorganisms-1263867-supplementary/Figure S13.tif]

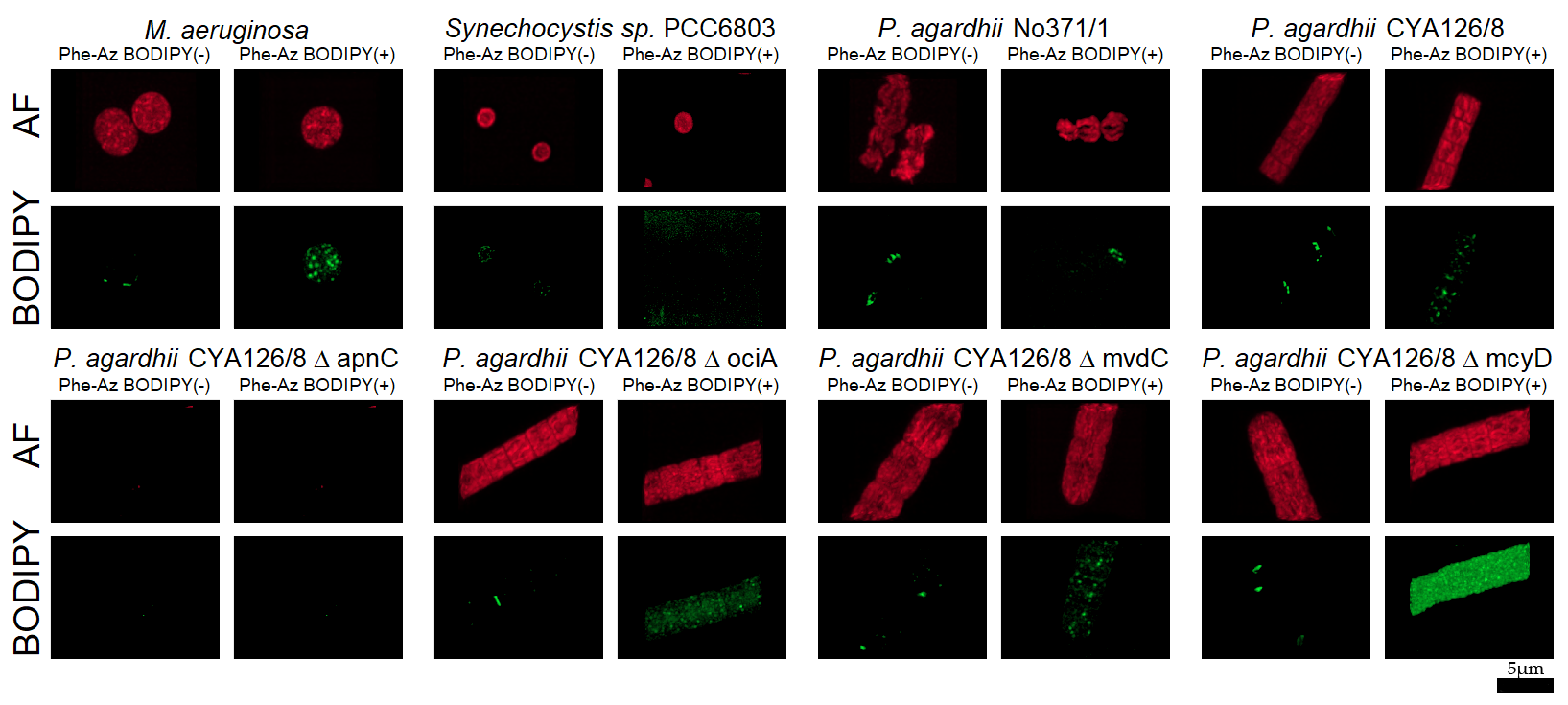

Supplement: Supplementary file 1 [file microorganisms-09-01578-s001.zip › microorganisms-1263867-supplementary/Figure S14.tif]

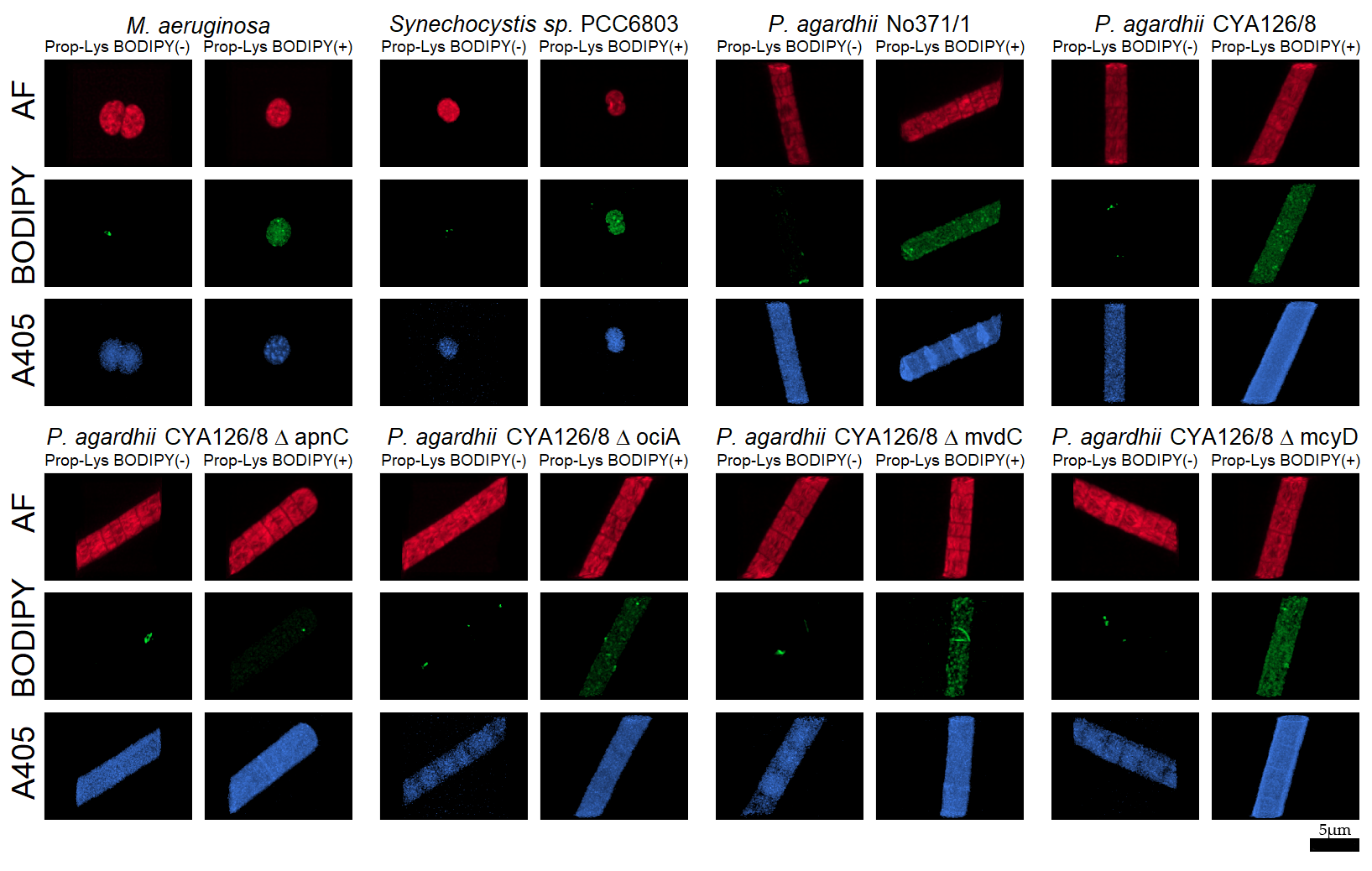

Supplement: Supplementary file 1 [file microorganisms-09-01578-s001.zip › microorganisms-1263867-supplementary/Figure S15.tif]

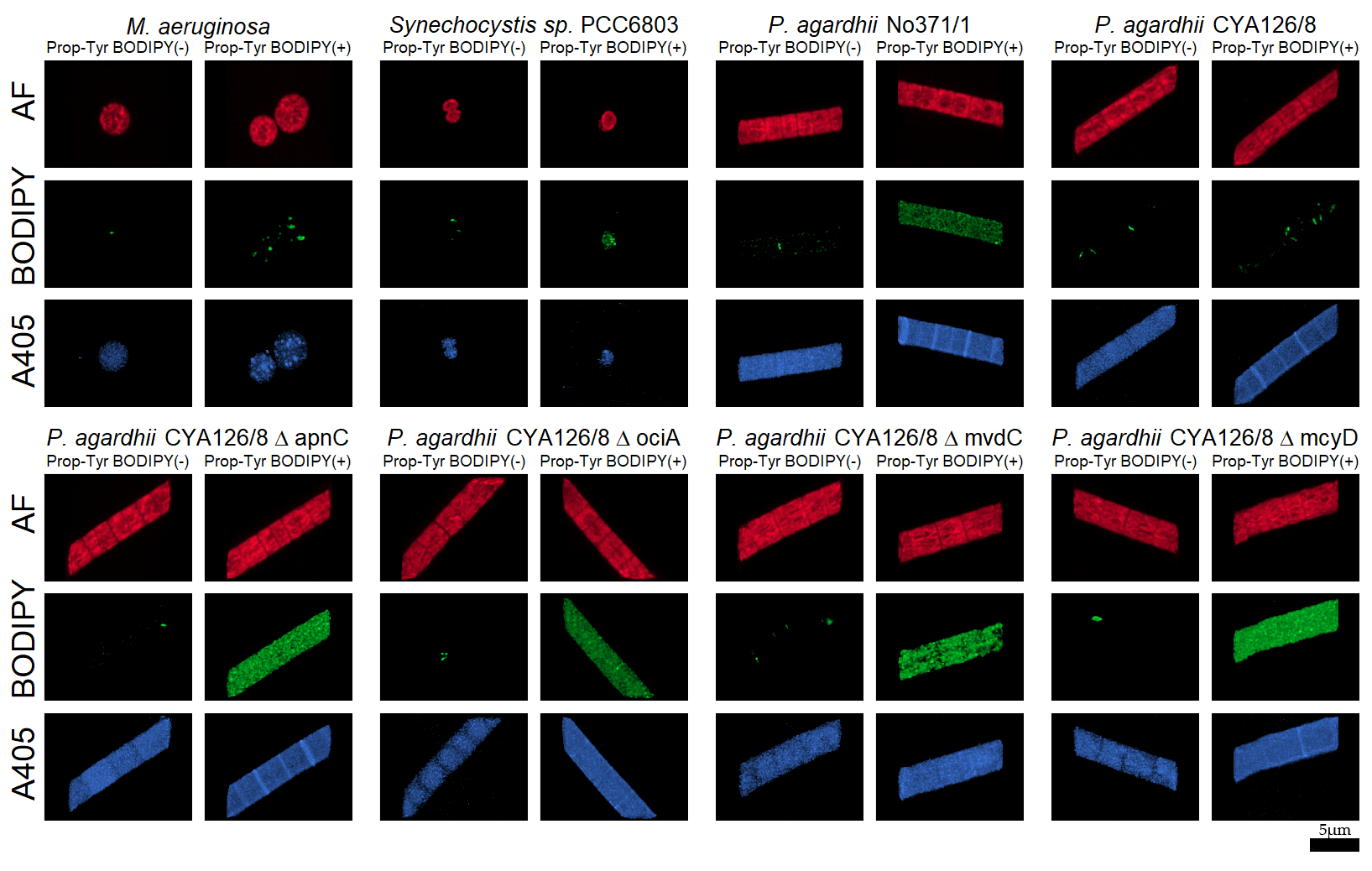

Supplement: Supplementary file 1 [file microorganisms-09-01578-s001.zip › microorganisms-1263867-supplementary/Figure S16.tif]

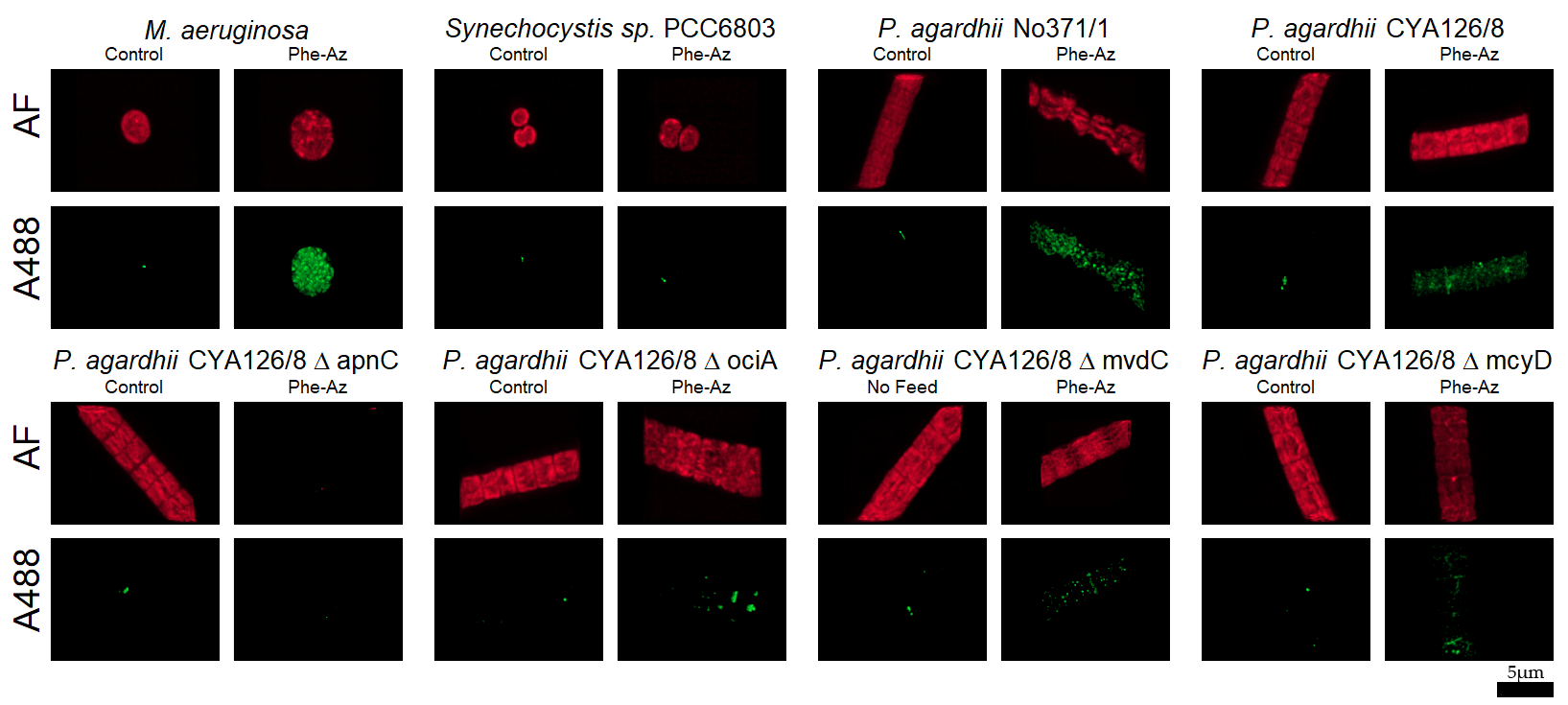

Supplement: Supplementary file 1 [file microorganisms-09-01578-s001.zip › microorganisms-1263867-supplementary/Figure S2.tif]

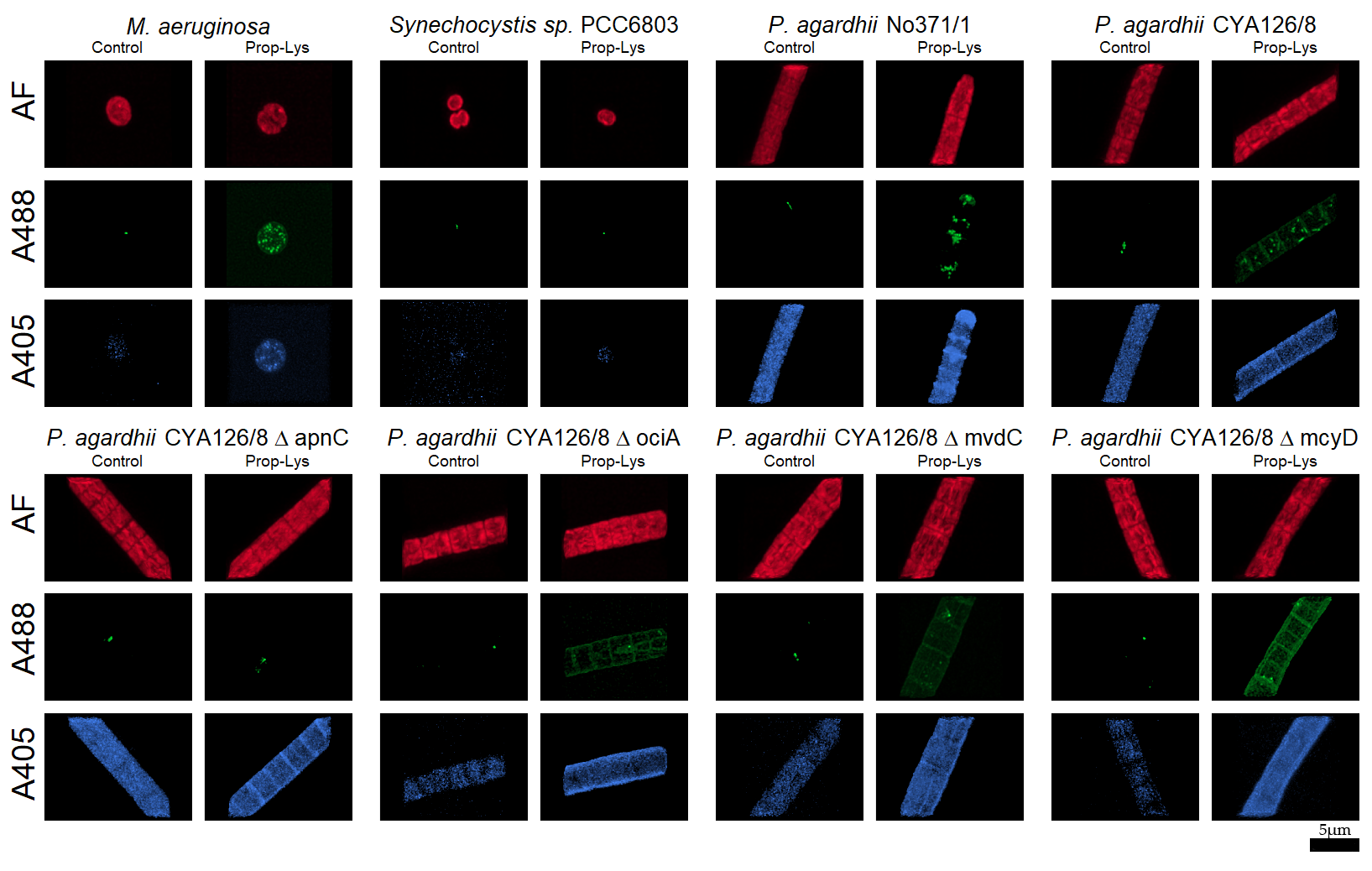

Supplement: Supplementary file 1 [file microorganisms-09-01578-s001.zip › microorganisms-1263867-supplementary/Figure S3.tif]

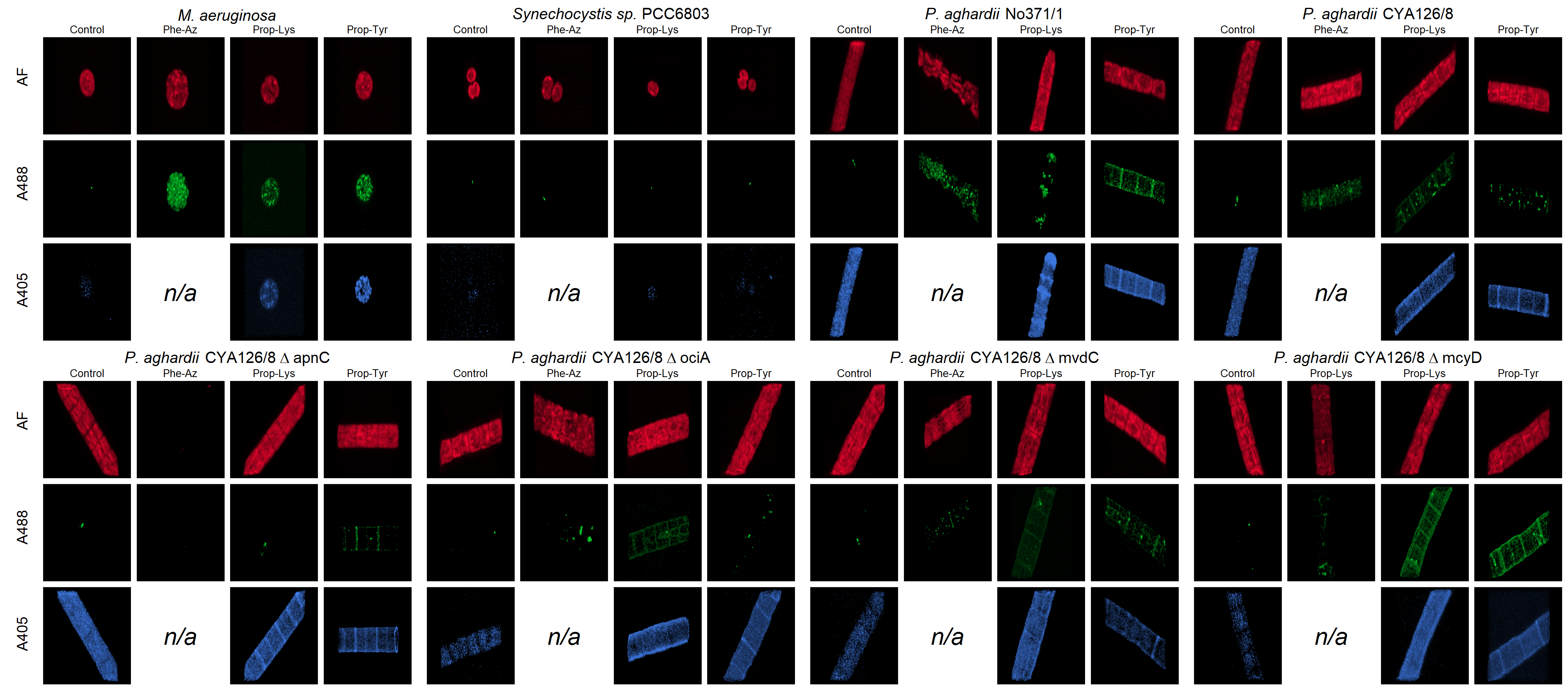

Supplement: Supplementary file 1 [file microorganisms-09-01578-s001.zip › microorganisms-1263867-supplementary/Figure S4.tiff]

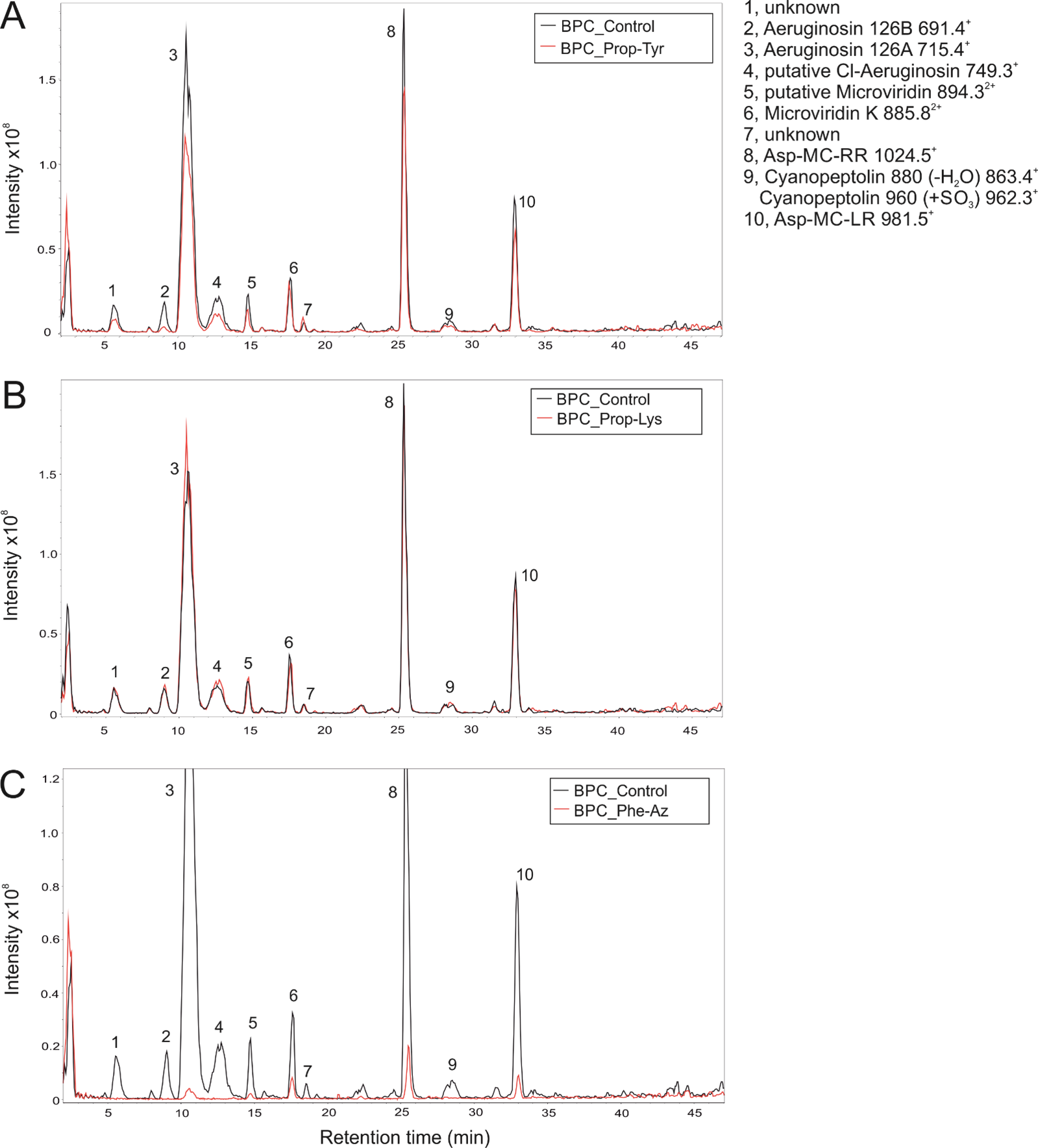

Supplement: Supplementary file 1 [file microorganisms-09-01578-s001.zip › microorganisms-1263867-supplementary/Figure S9.tif]
